# X-ray fluorescence detection for serial macromolecular crystallography using a JUNGFRAU pixel detector

**DOI:** 10.1107/S1600577519016758

**Published:** 2020-02-07

**Authors:** Isabelle Martiel, Aldo Mozzanica, Nadia L. Opara, Ezequiel Panepucci, Filip Leonarski, Sophie Redford, Istvan Mohacsi, Vitaliy Guzenko, Dmitry Ozerov, Celestino Padeste, Bernd Schmitt, Bill Pedrini, Meitian Wang

**Affiliations:** a Paul Scherrer Institute, Forschungsstrasse 111, Villigen 5232, Switzerland; b Center for Cellular Imaging and NanoAnalytics (C-CINA), Biozentrum, University of Basel, Basel 4058, Switzerland; c SwissNanoscience Institute, University of Basel, Basel 4056, Switzerland

**Keywords:** serial crystallography, macromolecular crystallography, fluorescence, JUNGFRAU detector, XFELs, silicon drift detectors

## Abstract

Detection of heavy elements, such as metals, in macromolecular crystallography (MX) samples by X-ray fluorescence is a function traditionally covered at synchrotron MX beamlines by silicon drift detectors, which cannot be used at X-ray free-electron lasers because of the very short duration of the X-ray pulses. Here it is shown that the hybrid pixel charge-integrating detector JUNGFRAU can fulfill this function when operating in a low-flux regime. The feasibility of precise position determination of micrometre-sized metal marks is also demonstrated, to be used as fiducials for offline prelocation in serial crystallography experiments, based on the specific fluorescence signal measured with JUNGFRAU, both at the synchrotron and at SwissFEL. Finally, the measurement of elemental absorption edges at a synchrotron beamline using JUNGFRAU is also demonstrated.

## Abstract 2

Use of the charge-integrating JUNGFRAU detector for fluorescence detection is investigated, and its applications in macromolecular crystallography at synchrotron and X-ray free-electron laser sources are demonstrated.

## 1. Introduction   

X-ray free-electron lasers (XFELs) are highly brilliant X-ray sources that deliver X-ray pulses with a duration of femto­seconds. These ultra-short pulses are attractive for macromolecular structure determination by crystallography because they allow the study of small crystals down to submicrometre sizes and give access to radiation-damage-free structures by the ‘diffract-before-destroy’ approach (Neutze *et al.*, 2000 ▸; Chapman *et al.*, 2011 ▸; Suga *et al.*, 2014 ▸). Because of the high peak intensity of the pulses, the sample is locally destroyed and data collection must be performed in a serial manner while replacing the sample between pulses (Schlichting, 2015 ▸). The XFEL beam time and protein samples are both extremely valuable, which means that both the hit rate (*i.e.* the proportion of useful images) and the sample consumption must be optimized. In fixed-target approaches, it is possible to reach high hit rates, either by prepositioning the crystals at specific positions (Mueller *et al.*, 2015 ▸; Oghbaey *et al.*, 2016 ▸; Roedig *et al.*, 2015 ▸; Opara *et al.*, 2017 ▸) or by prelocating the crystals on their support and addressing them at their positions with the beam in a precise manner (Cohen *et al.*, 2014 ▸).

In the crystal prelocation approach, the crystal positions are identified prior to diffraction data collection. Possible pre­location methods include X-ray based methods such as diffraction-based rastering (Wojdyla *et al.*, 2016 ▸) or X-ray imaging (Warren *et al.*, 2013 ▸; Wojdyla *et al.*, 2016 ▸), and optical imaging methods such as UV–visible fluorescence (Calero *et al.*, 2014 ▸), cross-polarization or second harmonic generation imaging (Madden *et al.*, 2013 ▸). The prelocation step can be performed online, meaning that the crystals are prelocated immediately before data collection without unmounting the sample from the goniometer or scanning stage used for data collection. This requires integration of the prelocation and diffraction data-collection setup. The prelocation step can alternatively be performed offline with a separate instrument, in which case the prelocated crystal coordinates must refer to fiducials on the sample support (Mueller *et al.*, 2015 ▸). The fiducials are then identified when the sample is mounted on the diffraction setup and data collection can take place based on the calculated crystal coordinates. The precise positioning of fiducials is therefore a key step on which the accuracy of the subsequent data-collection process relies. Positioning inaccuracies larger than the beam size would result in missing the crystals while locally damaging the sample with the XFEL beam. Positioning of fiducials, or more generally coordinate retrieval, at the beamline has been achieved by visual identification on the online viewing system (Sherrell *et al.*, 2015 ▸), either letting the user manually select them on the graphical user interface or using automatic pattern recognition schemes on the chip (Roedig *et al.*, 2017 ▸). However, in some samples the fiducials may be hardly visible. Manually indicating their position on the user interface, *e.g.* by mouse clicking, may be too imprecise or time-consuming and is not amenable to automation. We propose to employ metal fiducials placed on the chips to serve as coordinate references and use their fluorescence signal as a fiducial detection method at the beamline. The advantages of this approach are that the energy-specific fluorescence signal cannot be hidden by the protein sample and support, *i.e.* the marks can be detected even if they are not visible, and the precision of the position determination relies solely on the hardware precision. This method is also easily amenable to automation. A prerequisite is that the metal fiducials also appear in the prelocation step used to find the crystal positions offline so that coordinate matching can be performed. This is the case in particular in X-ray imaging methods, as we will separately show (manuscript in preparation).

The silicon drift detectors (SDDs) commonly used at synchrotron beamlines cannot serve at XFELs since they count single photons only at microsecond rates (Newbury, 2006 ▸). Spectral recording at XFELs is currently often carried out using specialized setups such as bent crystal spectrometers, *e.g.* in von Hamos geometry (Milne *et al.*, 2017 ▸; Kern *et al.*, 2015 ▸), or reflective zone plates (Kern *et al.*, 2015 ▸). The angular footprint of such spectrometers is not negligible so their integration in the measurement setup must be carefully planned and compromises must be found for collecting diffraction data simultaneously (Milne *et al.*, 2017 ▸; Kern *et al.*, 2015 ▸). The spectral resolution and performance offered by these instruments is higher than that required for certain applications such as simple detection of a known metal’s presence by fluorescence. However, bent crystal spectrometers certainly remain indispensable for other advanced macromolecular crystallography (MX)-related experiments requiring the detection of subtle signal modulations, for instance for the detection of oxidation states in protein-bound ions and functional clusters (Kern *et al.*, 2015 ▸). Energy-discriminating measurements have also been demonstrated at XFELs using pnCCDs (Strüder *et al.*, 2010 ▸; Hatsui & Graafsma, 2015 ▸).

The low noise and high sensitivity of modern hybrid pixel X-ray detectors make them good candidates for metal-detection applications. Hybrid pixel X-ray detectors are composed of a sensor, usually made of silicon for the conventional energy range for MX applications, directly bump-bonded to an application-specific integrated circuit (ASIC). Detectors with two classes of ASICs are available: photon counting and charge integrating. Photon-counting detectors increase a digital counter by one when the signal exceeds a defined threshold because of a photon hit. In the case of photon counters with a single threshold like PILATUS and EIGER (Dinapoli *et al.*, 2011 ▸), the pixel output corresponds to the number of events where the photon energy was higher than the defined threshold. This allows the suppression of dark and read-out noise and of lower-energy fluorescence by placing the threshold between the fluorescence energy and the incoming beam energy, though it does not allow for detecting only lower-energy fluorescence photons while ignoring higher-energy photons at the incoming beam energy. However, such a filtering of photons by energy could be achieved with photon counters having more than one threshold (color mode), *e.g.* Medipix3 (Ballabriga *et al.*, 2011 ▸) and EIGER2 (Bochenek *et al.*, 2018 ▸; Brönnimann & Trüb, 2018 ▸). But the photon-counting technology would remain inadequate for XFEL applications because of the pulse brilliance, because after counting a photon the electronic signal needs to decay below the threshold value before another photon can be detected, resulting in a ‘dead time’ and photon pile-up limitations. This is extremely severe for XFEL pulses, where the photon-counter dead time is orders of magnitude longer than the duration of a single pulse. In charge-integrating detectors such as JUNGFRAU, the total charge generated by the photons is collected during the integration time in each pixel and read out for each acquisition. This makes charge-integrating detectors optimal for XFEL applications, as the incoming photon number can be determined even if photons arrive within a short pulse. The pixel output is directly proportional to the energy of the incoming photons. If the flux is low enough that most pixels record single photons, individual photon energies can be readily measured. Alternatively, the number of photons can be determined as the total charge divided by the charge generated by a single photon.

**Figure 1:**
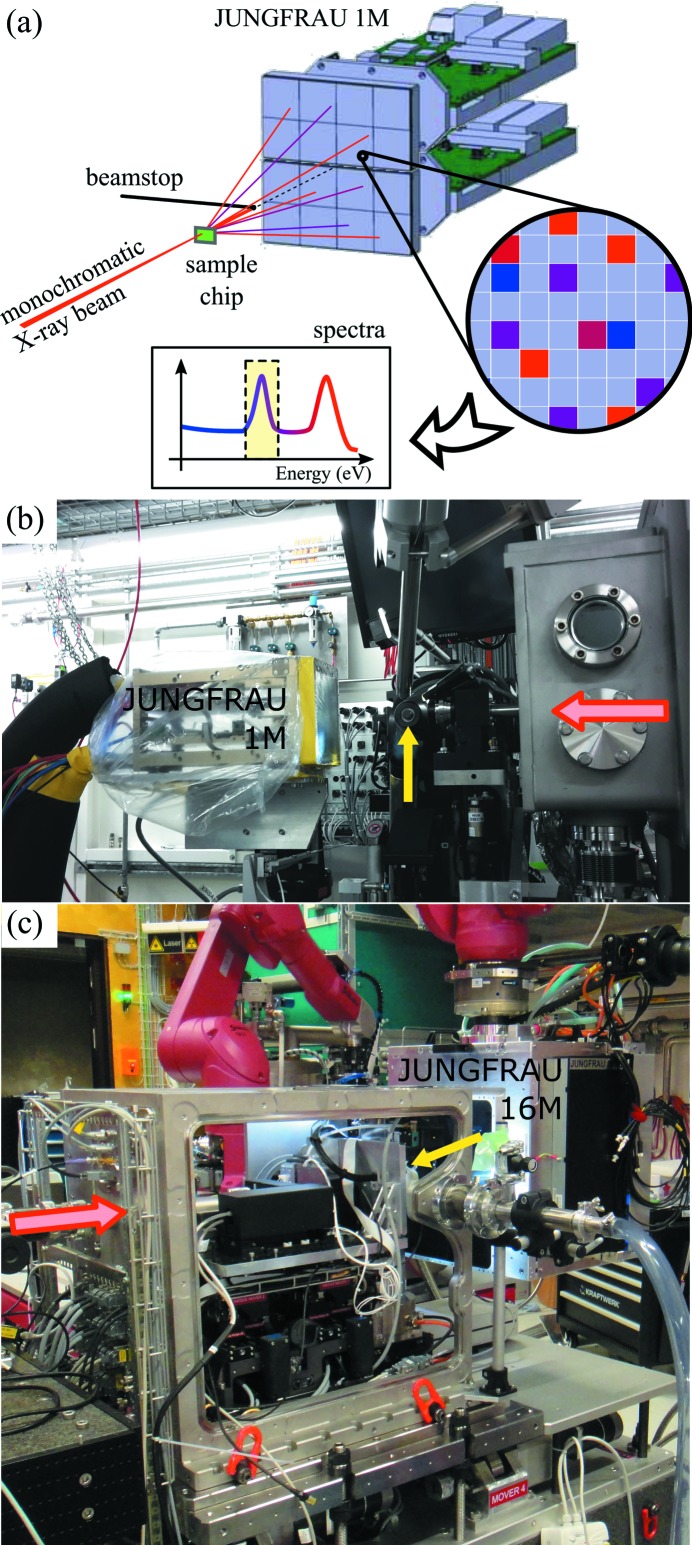
(*a*) Schematic of the geometry of the experiment and how spectra are obtained. (*b*) Photograph of JUNGFRAU 1M installed at the X06SA PXI beamline, with external cooling and nitro­gen environment. (*c*) Photograph of JUNGFRAU 16M in operation at SwissFEL Bernina with the SwissMX instrument. The red arrows show the direction of the X-ray beams and the yellow arrows show the sample positions.

Here we demonstrate practically that the JUNGFRAU hybrid pixel charge-integrating detectors that are installed at SwissFEL, among other facilities, are suitable for the detection of energy-dispersive fluorescence signals for two major fluorescence-based MX applications. We make use of the charge-integration technology in a regime of very low photon flux, and therefore very low dose, to determine the energy of single incoming photons on single pixels and combine the full detector area information into a fluorescence spectrum [Fig. 1 ▸(*a*)]. The obtained spectra provide a reliable fluorescence signal for finding micrometre-sized metal fiducials within model and representative protein-containing samples from scanning maps. We also show here that JUNGFRAU is suitable for recording fluorescence absorption-edge scans on standard samples, suggesting that it could fulfill the function traditionally devoted to SDDs at MX synchrotron beamlines. The application of JUNGFRAU for synchrotron crystallography data collection has been developed recently (Leonarski *et al.*, 2018 ▸).

## 2. Material and methods   

For concision, experimental details about the preparation of samples, data processing and absorption-edge scans are given in the supporting information. Fig. S13 in the supporting information is a schematic with the distances of the experimental setup.

### 2.1. Synchrotron data collection of fluorescence maps   

The JUNGFRAU 1 megapixel (1M) detector was installed at the X06SA PXI beamline at Swiss Light Source (SLS), Villigen, Switzerland [Fig. 1 ▸(*b*)]. It consists of two 0.5 megapixel modules stacked vertically, with a horizontal gap of 2.7 mm and total dimensions of 77 mm × 80 mm. The sensitive surface is protected by a 20 µm aluminized mylar film. External cooling was applied to reduce the noise level in long integration times, set at a temperature of −12°C. A protective plastic film filled with nitro­gen gas was wrapped around the detector to prevent condensation. The detector was placed on a stack of *xyz* stages, with the *z* stage (beam direction) motorized. The silicon sensor thickness was 320 µm. Pedestals were recorded for all datasets with the X-ray shutter closed and detector calibration was performed in the laboratory prior to the experiments (Redford *et al.*, 2018*a* ▸). The beamline energy was set to 12.398 keV. The beam size was 5 µm × 5 µm with a full flux of 1.13 × 10^11^ photons s^−1^. The sample-to-detector distance was 40 mm and the sample-to-beamstop distance was 15 mm.

Mapping was performed using the D3 goniometer (Fuchs *et al.*, 2014 ▸), similar to the rastering procedure described by Wojdyla *et al.* (2016 ▸). The grid-cell dimensions were identical to the beam size, 5 µm × 5 µm, except for oversampled scans where the cells were 2 µm × 2 µm and 1 µm × 1 µm. Unless stated otherwise, the beam transmission was 4% and the detector was triggered at the beginning of each row to collect images at a rastering rate of 100 Hz, while the detector was operated with a 1.0 kHz repetition rate and a duty cycle of 0.25 (*i.e.* 250 µs integration time per image), meaning that ten images were collected per cell of the raster grid.

Fluorescence maps were also collected with the photon-counting EIGER X 16M (Dectris, Baden-Daettwil, Switzerland) using the standard rastering procedure of the X06SA PXI beamline (Wojdyla *et al.*, 2016 ▸), using a single image per cell without changing the automatically set internal threshold of half of the incoming photon energy, which is below the fluorescence energy of the investigated metals for the incoming radiation of 12.389 keV. The detector distance for the EIGER 16M measurements was 135 mm.

### 2.2. Synchrotron data collection of absorption-edge scans   

The setup was essentially similar to that used for the fluorescence maps. Complete details are given in Section S2.1 of the supporting information. The JUNGFRAU 1M detector was operated at 2.2 kHz, since this operating condition had become available at the time of the measurement.

### 2.3. XFEL data collection of fluorescence maps   

Fluorescence data were also collected at the SwissFEL Bernina station (Ingold *et al.*, 2019 ▸), using the JUNGFRAU 16 megapixels (16M) operated at room temperature and mounted on a robot arm attached to the ceiling [Fig. 1(*c*) ▸]. The beam parameters were 9.06 keV photon energy, 250 µJ pulse energy (full beam) and 25 Hz repetition rate. The beam was focused slightly smaller than 5 µm × 5 µm FWHM and attenuated to typically 0.64% transmission. Samples were scanned using the SwissMX instrument for fixed-target MX (Milne *et al.*, 2017 ▸; Ingold *et al.*, 2019 ▸). The integration time of the detector was 10 µs. The sample-to-detector distance was 0.2 m and the entrance of the post-sample tube (2 mm outer diameter) was placed 25 mm after the sample. Each pulse was recorded in a separate image and corresponds to a unique scanning position and grid cell, in contrast to the SLS experiment where several images were summed for each cell of the grid.

## 3. Results and discussion   

### 3.1. Choice of data-collection parameters   

The data-collection parameters were carefully chosen to guarantee that the number of incoming photons per pixel per frame was well below 1, both at the synchrotron and at the XFEL. This is necessary to ensure that the maximum charge detected by each pixel corresponds to an isolated photon so that the final spectrum reflects the incoming radiation as accurately as possible. In these low-flux conditions, double counts are minimized; however, charge-sharing effects between neighboring pixels are still present. A typical image contained more than 90% of pixels with no apparently detected photon (Fig. S9). The suitable range of conditions corresponds in practice to several orders of magnitude attenuation of the X-ray beam, which are realistic parameters for XFELs in normal self-amplified spontaneous emission (SASE) mode or using a monochromator.

Another important parameter is the dose received by the protein samples. With the typical beam parameters used here for collecting fluorescence maps with JUNGFRAU, the dose received by a crystal with the same size as the beam is of the order of 1 kGy. This dose is two orders of magnitude lower than the dose received by the same crystal during a standard diffraction rastering at 100 Hz with full beam, which is about 0.1 MGy. In the absorption-edge measurements at the synchrotron, the exposure conditions were comparable with those of an SDD measurement.

### 3.2. Fluorescence maps   

#### 3.2.1. Scanning maps on model samples   

**Figure 2:**
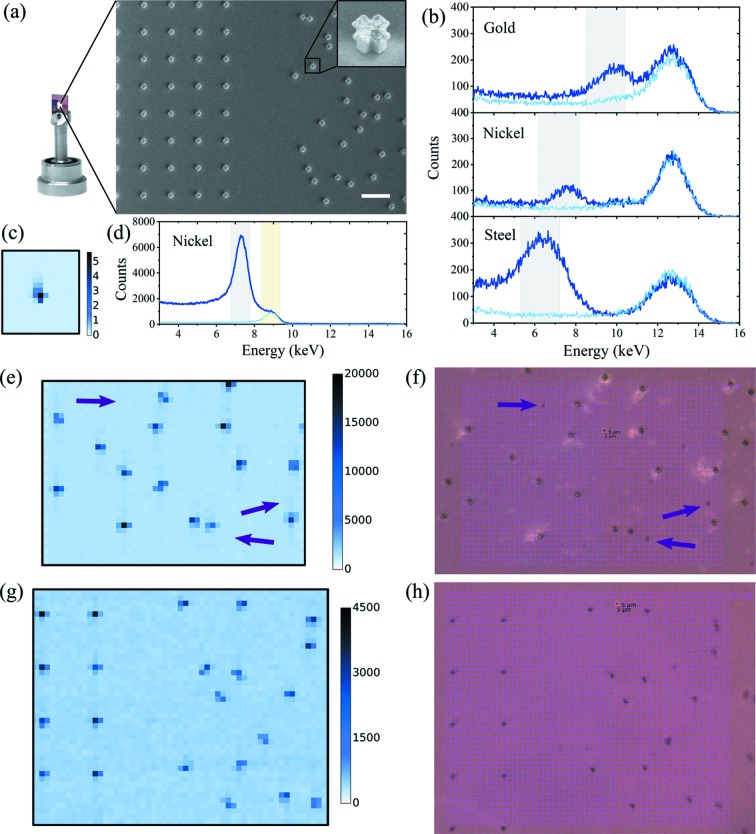
(*a*) Si_*x*_N_*y*_ membrane fixed on a chip pin and a scanning electron microscopy (SEM) image of the electroplated metal structures on the Si_*x*_N_*y*_ membrane; the scale bar is 20 µm. Inset: magnified view on a 3 µm gold cross with a 35.6° tilt. (*b*) and (*d*) Representative spectra, cumulated from all detector pixels, for different metals investigated: (*b*) from representative samples sandwiched between Si_*x*_N_*y*_ windows at the SLS (JUNGFRAU 1M) and (*d*) from a model nickel sample at SwissFEL (JUNGFRAU 16M). The thick dark blue curves correspond to a grid cell where the metal was present; the thin light blue curves correspond to another cell of the same grid where the metal was absent. The gray areas represent the ROI used for extracting the fluorescence signal. (*c*) and (*e*)–(*h*) Examples of maps obtained from gold [(*e*) and (*f*)] and nickel [(*c*), (*g*) and (*h*)] model samples. (*c*) Fluorescence map from a JUNGFRAU 16M at the SwissFEL. The color scale shows the ratio of fluorescence to direct-beam ROI counts. For this FEL case, the signal from the fluorescence ROI was normalized to the counts in the direct-beam energy ROI [shown as gray and yellow, respectively, in (*d*)]. (*e*) and (*g*) Fluorescence maps from a JUNGFRAU 1M at the SLS. The corresponding inline camera view is shown in (*f*) and (*h*). The purple arrows point to dust particles present on the membranes. The color scales show summed ROI counts (arbitrary unit of fluorescence signal).

Fluorescence maps were first measured at the SLS by raster-scanning samples on a regular grid at very low incident flux and summing the pixels which recorded a photon with an energy around the expected metal fluorescence energy [Fig. 1 ▸(*a*)]. Map scanning was initially performed on model samples carrying only metal marks of 1 µm thickness, consisting of 3 µm-sized crosses with 1 µm branches [Fig. 2 ▸(*a*)]. Representative spectra are shown in Fig. 2 ▸(*b*) and Fig. S4 of the supporting information. The peak at the incoming photon energy is present in all curves and results from elastic scattering from the direct beam. A second peak at the fluorescence energy of the metal is visible in the presence of the metal (dark blue curves). All the spectra also display a charge-sharing baseline signal extending from the highest incoming energy down to zero (see Section 3.2.3). Fig. 2 ▸ shows representative maps obtained from the Au and Ni samples [Figs. 2 ▸(*e*) and 2(*g*)] with a comparison with the online microscope view where the grid has been defined [Figs. 2 ▸(*f*) and 2(*h*)]. With the fluorescence maps, metal dots are easily distinguished from dust particles [purple arrows in Figs. 2 ▸(*e*) and 2 ▸(*f*)] deposited on the membrane surface, which are present in the optical image but do not give rise to a fluorescence signal at the expected energy. The signal-over-background ratio (SBR) for the mapping scans presented in Figs. 2 ▸(*e*) and 2(*g*) is 10.2 ± 0.2 and 7.3 ± 0.2 for Au and Ni, respectively.

After this successful proof-of-principle demonstration at the SLS, the viability of the detection was confirmed at SwissFEL. Fluorescence map scans were performed at SwissFEL during commissioning time, using the SwissMX fixed-target station (Milne *et al.*, 2017 ▸; Ingold *et al.*, 2019 ▸) and JUNGFRAU 16M of the Bernina endstation, on the nickel model samples. Because of time limitations, only a few scans were recorded and only on small areas around individual marks. Fig. 2 ▸(*c*) shows a scan over a single nickel mark and Fig. 2 ▸(*d*) shows the corresponding representative spectra with a SBR of 21.5. The enhanced SBR at SwissFEL compared with the SLS seems to result from the relatively lower intensity of the direct-beam scattering, which may come from subtle differences in the measurement endstations (Fig. S13), such as the beamstop or post-sample tube size and distance to the sample, as well as the slightly different beam sizes. The higher counts read in Fig. 2 ▸(*d*) compared with Fig. 2 ▸(*b*) results from a factor 50 in the incoming flux between both setups.

The SwissMX scanning stages can be enclosed in a chamber for operation in a helium or air environment. In helium, low-energy X-rays such as fluorescence signals from Fe or Ni may be detected more easily since they are not absorbed in the long path in air until they reach the detector. However, this effect will be partly compensated by the kapton back-window closing the He chamber, as well as the possibly increased thickness of the protective aluminized kapton foil on the detector.

#### 3.2.2. Influence of collection parameters on model samples   

**Figure 3:**
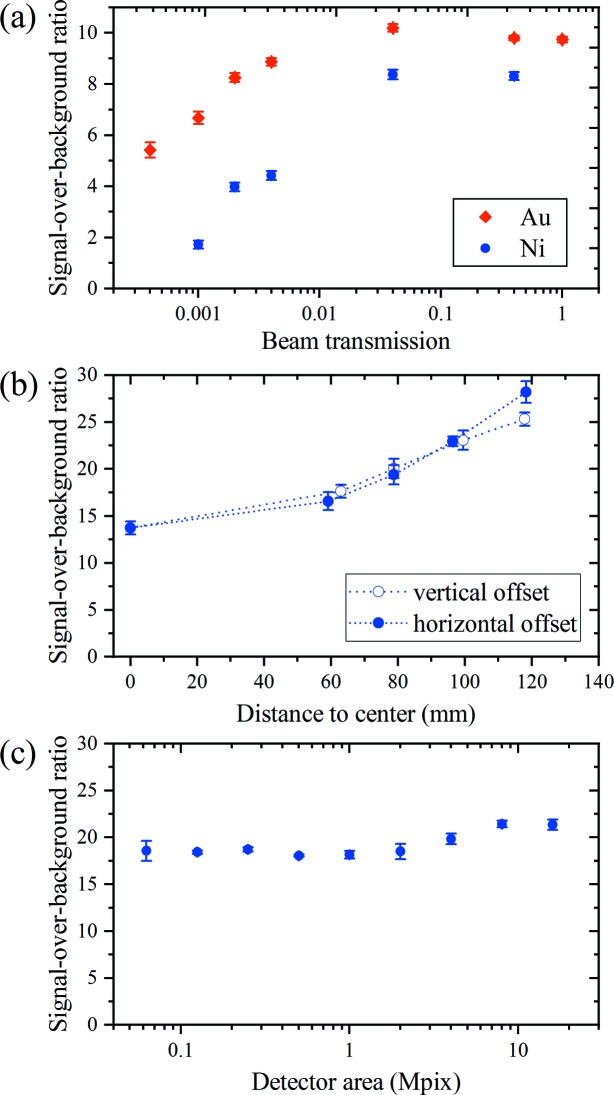
(*a*) SBR as a function of the beam transmission for gold and nickel model samples measured at the SLS with a JUNGFRAU 1M. (*b*) SBR from a nickel model sample as a function of the vertical and horizontal offset of a 1M spatial ROI on the JUNGFRAU 16M at SwissFEL. (*c*) SBR from a nickel model sample as a function of the area of the spatial ROI on the JUNGFRAU 16M at SwissFEL.

A systematic study of several parameters was undertaken to evaluate the robustness of the detection method. At the synchrotron, the X-ray beam transmission was varied between 0.04% and 100% to investigate the influence of the number of photons per exposure [Fig. 3(*a*) ▸]. An optimal transmission was observed because of the competition between increased background noise at low transmission and progressive departure from the conditions of a single photon per pixel at high transmission, where the energy of the incoming photons can no longer be determined reliably. Representative maps are shown in the supporting information (Fig. S5). Table 1 ▸ shows the SBR obtained while varying other collection parameters on the same grid. The duty cycle was compared at 0.25 and 0.01 to investigate detector noise levels. In this case, a moderate loss of SBR is observed with the longer integration time because of the accumulation of noise. The rastering speed was compared at 10 Hz and 100 Hz while keeping the number of photons constant. A slightly lower fluorescence signal was observed at the lower rastering speed (Fig. S5), which caused a moderate drop in SBR. This might be caused by imprecision in the transmission settings or by a more precise sampling of the metal mark with the partial duty cycle used here. Generally, situations where the overall number of incoming photons is reduced give noisier spectral curves (*cf*. Fig. S6). However, within a certain range of variation of experimental parameters, the SBR obtained from the summed region of interest (ROI) counts remains relatively unchanged and reliable. This indicates that the detection technique is robust and could be employed over a wide range of measurement conditions.

In the data collected at SwissFEL, the large area of the 16M detector makes it possible to study the effects from the positioning and area of the sensitive surface. In Fig. 3 ▸(*b*), the fluorescence was measured on square 1M spatial ROIs placed on the 16M area with offsets to the center in the vertical and horizontal directions [Fig. S11(*a*)]. The SBR increases with the offset to the center because the fluorescence photons are scattered essentially isotropically and therefore are overrepresented compared with elastic scattering which takes place predominantly in the forward direction. On the edge of the detector, the two curves slightly separate: the fluorescence counts and SBR become higher for a horizontal offset than for a vertical offset, possibly reflecting the polarization of the X-ray beam. Fig. 3 ▸(*c*) shows that the detected signal essentially does not depend on the area of the detector over the wide range studied. The rectangular ROIs were chosen to preserve comparable radial symmetries [Fig. S11(*b*)] in order to eliminate the angular effects presented in Fig. 3 ▸(*b*). This becomes difficult at large areas, where high-angle pixels are slightly overrepresented, thus increasing slightly the measured SBR above 2 megapixels.

For comparison, scanning maps were also performed at the synchrotron using a detector without energy-dispersive detection features, *i.e.* a large-area single-photon-counting detector EIGER 16M (Table 1 ▸), using as close as possible parameters (see Section 2), in particular the same solid angle and incoming flux per cell. The obtained fluorescence SBRs are substantially lower and are strongly influenced by the integration geometry (Fig. S12). In particular, in the forward direction (*i.e.* when integrating close to the direct beam), the SBR is so low that fluorescence detection becomes impossible with EIGER [Fig. S12(*a*)], whereas the SBR remains high with JUNGFRAU [Fig. 3 ▸(*b*)].

#### 3.2.3. Scanning maps on real-life samples   

To assess the practical usefulness of the method, it is required to evaluate the impact of the presence of other materials in the beam path, which increases the elastic signal by scattering the incoming X-rays and reduces the SBR. The real-life protein-containing samples investigated here are representative of *in situ* methods (Aller *et al.*, 2015 ▸; Martiel *et al.*, 2018 ▸) where the matrix or mother liquor is not removed from the support, thus resulting in a high elastic scattering signal. In other experimental methods, it is possible to remove the mother liquor to decrease the background and to improve the diffraction SBR (Roedig *et al.*, 2015 ▸; Oghbaey *et al.*, 2016 ▸). The fluorescence signal from such samples would also benefit from the mother-liquor removal and resemble more the SBR obtained from model samples. The choice of high-background real-life protein-containing samples in our experiments exemplifies the applicability of the method presented here to a broad range of samples.

In the scope of this work on fluorescence detection, no diffraction data were collected from the embedded protein crystals. Demonstration of MX data collection from prepositioned protein crystals using fiducials on the same samples will be the topic of a separate publication. Characterization of the MX data-collection performance of JUNGFRAU has been reported elsewhere (Leonarski *et al.*, 2018 ▸). The fluorescence signal is used solely to detect the metal fiducials and never to detect the crystals themselves, as the offline prelocation process implies that these are detected using a different technique.

**Figure 4:**
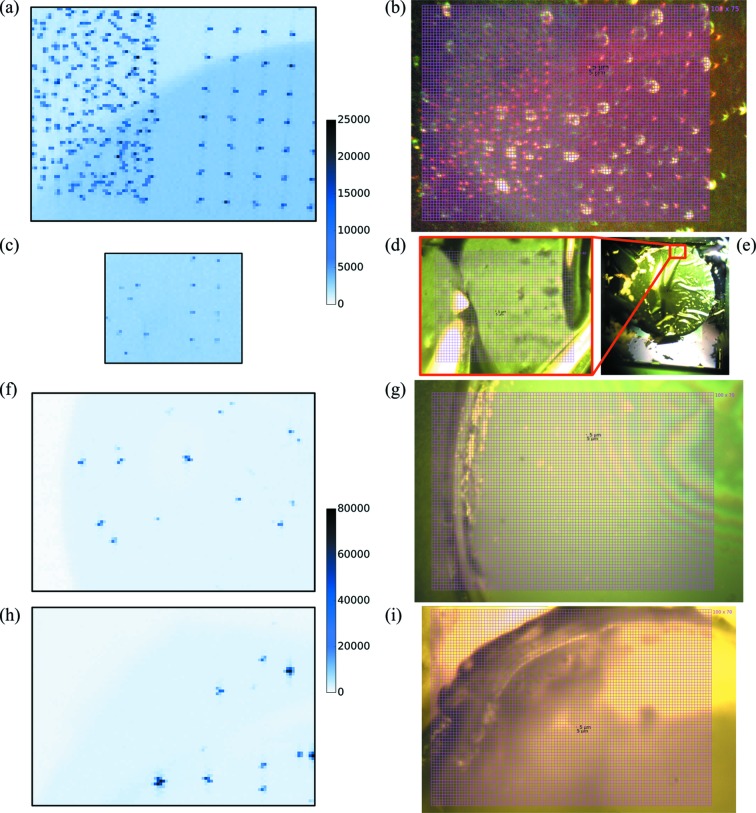
JUNGFRAU 1M fluorescence maps from real-life protein-containing samples [(*a*), (*c*), (*f*) and (*h*)] with corresponding views on the online camera [(*b*), (*d*), (*e*), (*g*) and (*i*)]. (*a*) and (*b*) Au marks. (*c*)–(*e*) Ni marks, where the Si_*x*_N_*y*_ membrane was partially destroyed around the LCP bolus. (*e*) A low-magnification view. (*f*) and (*g*) Steel microbeads embedded in LCP within Si_*x*_N_*y*_ sandwich and (*h*) and (*i*) within COC sandwich. Maps (*a*) and (*c*), and (*f*) and (*h*) are plotted in the same scale of summed ROI counts.

Two types of representative real-life samples containing protein crystals were investigated. In the first sample, the micrometre-thick Au or Ni fluorescent marks on Si_*x*_N_*y*_, previously investigated as model samples, were used as one side of a 140 µm sandwich of lipidic cubic phase (LCP) containing protein crystals [Figs. 4 ▸(*a*)–4(*e*)] to determine whether the uniformly spaced marks could still be detected in the presence of increased background. In the second sample, steel microbeads of 1 to 22 µm diameter were randomly embedded in the crystal-containing LCP to simulate iron fluorescent marks with unknown spacing and sandwiches were prepared from both Si_*x*_N_*y*_ membranes without metal marks [Figs. 4 ▸(*f*) and 4(*g*)] and 25 µm-thick cyclic olefin copolymer (COC) films [total thickness 190 µm, Figs. 4 ▸(*h*) and 4(*i*)]. In all cases, the positions of metal objects were readily identified in the fluorescence map, while they were not always clearly visible in the online camera view, for instance inside the frozen bolus. The bolus edges clearly appeared as a drop in the background counts. For the Si_*x*_N_*y*_ sandwich with gold and nickel marks, the SBR was 2.6 ± 0.1 and 2.5 ± 0.1 respectively, compared with 10.2 ± 0.2 and 7.3 ± 0.2, respectively, for the model samples measured in identical conditions. The drop in SBR is caused by the higher absorption and signal from X-ray scattering in the LCP matrix. For the steel beads in Si_*x*_N_*y*_ and COC sandwiches, the SBR was 16.0 ± 0.2 and 29.3 ± 0.1, respectively. The SBR values for the steel beads cannot be directly compared with the gold and nickel cases because the steel beads are much larger than the thickness of the microfabricated gold and nickel patterns. With the EIGER 16M, the fluorescence could not be readily detected on the same real-life protein-containing samples (Fig. S10).

Hybrid pixel detectors display charge-sharing effects, *i.e.* when a photon hits the edge or the corner of a pixel the energy deposited is collected by up to four pixels. Because of this charge-sharing effect, charge-integrating hybrid pixel detectors always provide a distribution of energies based on single pixels which extends below the true photon energy all the way to zero as a continuous energy background in the spectrum, in addition to the main energy peak. Charge summation of neighboring pixels is often used to obtain a correct energy distribution, albeit at the cost of a higher noise level. In order to assess the potential for improvement of SBR, clustering analysis was performed on the Si_*x*_N_*y*_ gold protein-containing-sample scan (Fig. S6). By summing energy readouts of clusters of neighboring pixels instead of considering energy readouts of individual pixels, the low energy baseline of counts induced by charge sharing is strongly reduced. This results in an improvement of the SBR, from 2.6 ± 0.1 to 5.7 ± 0.1 on this fluorescence map.

#### 3.2.4. Precision of position determination   

**Figure 5:**
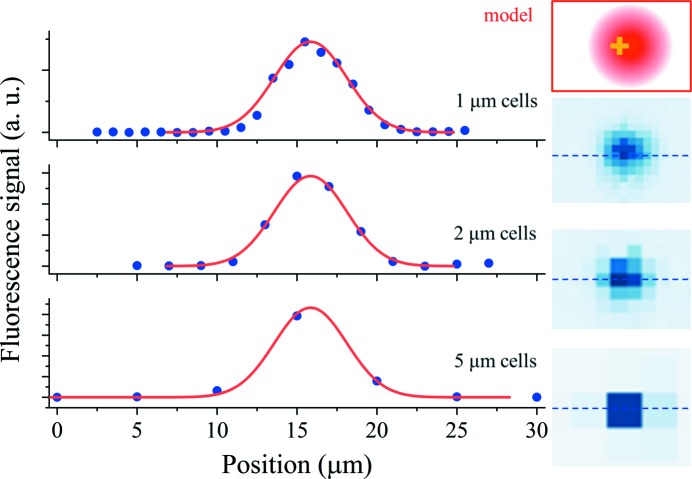
Cross section of a gold cross fluorescence map with (from top to bottom) 1 µm, 2 µm and 5 µm cell size, showing the experimental (blue dots) fluorescence profile and fitted overlap model (red curve). The horizontal slices on the left panel correspond to the dashed blue lines on the fluorescence maps on the right panel. Framed inset (top right), schematic of the overlap model used for modeling the experimental signal: convolution of the 3 µm-wide cross shape and the 5 µm FWHM Gaussian beam.

For high hit-rate data collection on prelocated samples, the fiducial marks must be precisely located. To assess the potential for accurate mark position determination, scans were also performed on the same samples (Figs. 2 ▸ and 4 ▸) in oversampled conditions, where the grid-cell dimensions were reduced compared with the beam size. This resulted in a slower scanning speed but more precise sampling of the fluorescence profile of the mark with overlapping frames. Fig. 5 ▸ shows map details and one-dimensional sections of the oversampled maps (blue dots), and examples of complete oversampled maps are given in Fig. S6. In order to determine the position of the center of mass of the metal marks, a theoretical fluorescence profile was calculated by convoluting the beam Gaussian intensity profile with the known shape of the mark, using a simple overlap model (Fig. 5 ▸, framed inset): the convolution of the 3 µm-wide cross shape and the 5 µm FWHM Gaussian beam was performed by summing the intersection volume while displacing the two objects relative to each other. The center of mass of the marks was then determined by fitting the theoretical curves to the experimental curves (Fig. 5 ▸, red curves) in both horizontal and vertical directions. A good agreement was obtained between the experimental and the theoretical profiles. For prelocation processes, distances are a relevant measurement because the spacings between fiducials matter more than their individual absolute positions. The quality of the position determination was assessed by measuring all distances between marks on a map and comparing with the expected values of the pattern design. Statistics were calculated on the values of the differences between measured and expected distances, taking the absolute differences to avoid error-compensation effects (Table 2 ▸). From these results, we concluded that the achieved precision of position determination is in all cases better than the size of the grid cells. Oversampling increases the position-determination precision, which makes it possible to reach submicrometre precision with a moderate oversampling. The lower SBR obtained from protein-containing real-life samples decreases the position-determination precision. The precision observed with the nickel protein-containing sample is worse than expected. As seen in Figs. 4 ▸(*d*) and 4(*e*), this sample was broken around the bolus and the mark-bearing membrane was wrinkled, which may partly explain the worsening of the precision of position determination.

### 3.3. Absorption-edge scans   

**Figure 6:**
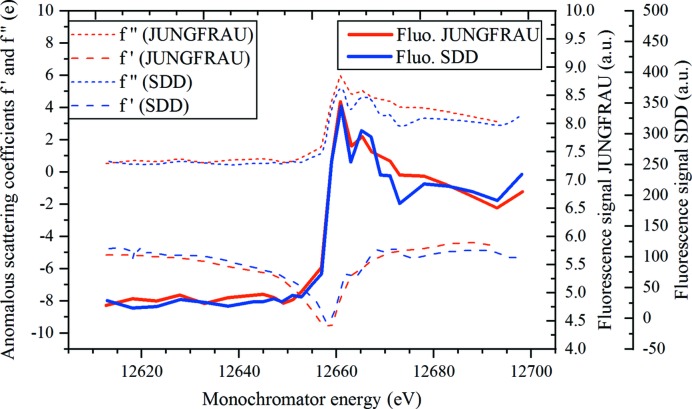
Edge scan over the Se edge on a 90 µm × 50 µm crystal showing a comparison between JUNGFRAU and an SDD measurement.

Proof-of-principle absorption-edge scan measurements were performed at the SLS on large protein crystals representative of standard synchrotron rotation experiments (Fig. 6 ▸). Experimental methods and results are detailed and discussed in the supporting information (Section S2, Fig. S8, Tables S1 and S2). A 90 µm × 50 µm crystal of seleno­methio­nine containing protein was chosen as an example displaying a clear white line above the absorption edge. Experimentally determined curves (Fig. 6 ▸) and values (Table S1) are in excellent agreement with the measurement performed with SDDs on the same sample, and within the variation observed between SDD measurements at two different beamlines [Fig. S8(*d*)]. The example of a mercury-soaked model crystal, an element for which the peak is not prominent, is also shown in the supporting information [Figs. S8(*c*) and S8(*e*), Table S2].

A charge-integrating pixel detector like JUNGFRAU, with small pixels and a relatively high noise, is arguably no match for a dedicated energy-dispersive detector like a commonly used SDD. Charge sharing produces a continuous background at all energies below the measured energy and the higher electronic noise gives photon-energy peaks which are a factor of five broader compared with peaks typically obtained with a SDD [Fig. S8(*a*)].

However, in the scope of this work, it is important to note that, in spite of this reduced spectrum quality, JUNGFRAU provides enough information to reliably retrieve edge scans of sufficient quality for the investigated purposes. A main JUNGFRAU detector installed at a synchrotron beamline could in principle also take over the function of a fluorescence detector for the applications presented above, thus freeing space in the crowded sample environment in MX beamlines. In addition, the high-angle scattering recorded in a typical diffraction image in MX experiments carried out in a low-dose and fine-phi slicing manner is usually very weak. Therefore, when JUNGFRAU is used as the main X-ray detector at an MX beamline, an analysis of high-angle background scattering could in principle be used to detect most bio-metals. In a multiwavelength anomalous dispersion (MAD) experiment, such utility could provide continuous monitoring of the beam energy and detect potential energy drifts around the absorption edge of interest throughout the entire data collection.

Regarding the possibility of measuring absorption edges at XFELs, it is important to note that the recording of absorption-edge scan data at the XFEL would require reducing the energy bandwidth of the XFEL beam by using a monochromator or a seeded injection scheme. XFEL SASE beams are characterized by a certain energy spread varying from pulse to pulse (Karvinen *et al.*, 2012 ▸; Tono *et al.*, 2013 ▸; Zhu *et al.*, 2012 ▸). The pulse-by-pulse monitoring of the incoming energy spectrum would also need to be included in the data analysis in order to preserve the quality of the resulting fluorescence spectra (Juranić *et al.*, 2018 ▸).

## 4. Conclusions   

We have practically demonstrated that the hybrid pixel charge-integrating detector JUNGFRAU can fulfill the functionalities of a spectrometer for X-ray fluorescence detection applications in macromolecular crystallography, thanks to its native energy-dispersive capabilities. The method presented here is valid over a wide range of beam attenuation, allowing low-dose fluorescence detection and therefore protein sample preservation. Two different MX-related applications were investigated: the accurate spatial detection of micrometre-sized metal marks in both model and representative protein-containing samples, and absorption-edge scans for anomalous data collection. The wide variety of metals used proves the versatility and robustness of the detection. In the case of fluorescence maps, sub-cell precision was obtained by fitting a theoretical fluorescence profile.

Our results are of practical importance considering that the SDDs commonly employed at synchrotron MX beamlines cannot be used at XFELs, whereas JUNGFRAU detectors are permanently installed or available on request at a number of facilities including SwissFEL. The energy-dispersive capacities of charge-integrating detectors such as JUNGFRAU could be useful in other types of imaging experiments where the energy-dispersion requirements are similar.

## 5. Related literature   

The following references are cited in the supporting information for this article: Brun & Rademakers (1997) ▸; Edelman *et al.* (1979) ▸; Evans & Pettifer (2001) ▸; Gorelick *et al.* (2010) ▸; Hili *et al.* (2015) ▸; Huang *et al.* (2016) ▸; Löbl & Huppertz (1998) ▸; Redford *et al.* (2018*b*) ▸; Zeldin *et al.* (2013) ▸.

## Supplementary Material

Materials and Methods, edge scan detailed information, and various supporting figures and tables (edge scan values). DOI: 10.1107/S1600577519016758/ig5086sup1.pdf

## Figures and Tables

**Table 1:** Influence of various detection parameters on the SBR for the gold and nickel model samples, measured at the SLS n.d = not determined. The duty cycle is the ratio between the integration time and period associated with the detector frequency of operation.

	Varying parameter	Experimental conditions	SBR (Au)	SBR (Ni)
Duty cycle	0.01	100% T, 100 Hz	11.4 ± 0.1	n.d.
	0.25	100% T, 100 Hz	9.7 ± 0.1	—
Raster speed	10 Hz (100 images cell^−1^)	0.4% T, duty cycle 0.25	6.6 ± 0.1	n.d.
	100 Hz (10 images cell^−1^)	4% T, duty cycle 0.25	10.2 ± 0.2	—
Detector type	JUNGFRAU 1M (energy dispersive)	4% T, 100 Hz, duty cycle 0.25	10.2 ± 0.2	7.3 ± 0.2
	EIGER 16M (total photon counts)	0.1% T, 10 Hz, duty cycle 1	4.2 ± 0.1	1.8 ± 0.2

**Table 2:** Effect of oversampling on the differences between the measured and expected distances between marks, in model and real-life protein-containing samples

		Au		Ni	
	Number of distances measured	Average absolute difference (µm)	Standard deviation (µm)	Average absolute difference (µm)	Standard deviation (µm)
1 µm cells, model sample	≥3	0.49	0.17	0.28	0.20
2 µm cells, model sample	≥10	0.60	0.41	0.45	0.26
5 µm cells, model sample	≥10	0.82	0.86	1.58	1.24
5 µm cells, real-life sample	≥10	1.31	1.49	2.24	1.64
