# Supplementary material for: X-ray fluorescence detection for serial macromolecular crystallography using a JUNGFRAU pixel detector
Source: J Synchrotron Radiat. 2020 Feb 7;27(Pt 2):329–39. doi: 10.1107/S1600577519016758 (PMC7064105; doi:10.1107/S1600577519016758)
Supplement: Supplementary file 1 [file s-27-00329-sup1.pdf]

JOURNAL OF
SYNCHROTRON
RADIATION

Volume 27 (2020)

Supporting information for article:

X-ray fluorescence detection for serial macromolecular crystallography using a JUNGFRAU pixel detector

Isabelle Martiel, Aldo Mozzanica, Nadia L. Opara, Ezequiel Panepucci, Filip Leonarski, Sophie Redford, Istvan Mohacsi, Vitaliy Guzenko, Dmitry Ozerov, Celestino Padeste, Bernd Schmitt, Bill Pedrini and Meitian Wang

X-ray fluorescence detection for serial macromolecular crystallography using a JUNGFRAU pixel detector

Authors

Isabelle Martiel[a]*, Aldo Mozzanica[a], Nadia L. Opara[abc], Ezequiel Panepucci[a], Filip Leonarski[a], Sophie Redford[a], Istvan Mohacsi[a], Vitaliy Guzenko[a], Dmitry Ozerov[a], Celestino Padeste[a], Bernd Schmitt[a], Bill Pedrini[a] and Meitian Wang[a]*

[a] Paul Scherrer Institute, Forschungsstrasse 111, Villigen, 5232, Switzerland

[b]Center for Cellular Imaging and NanoAnalytics (C-CINA), Biozentrum, University of Basel, Basel, 4058, Switzerland

[c]SwissNanoscience Institute, University of Basel, Basel, 4056, Switzerland

Correspondence email: isabelle.martiel@psi.ch; meitian.wang@psi.ch

Supporting information

S1. Materials and methods for the fluorescence maps experiments

S1.1. Fabrication of gold and nickel model structures by electroplating

Metal marks of 1 and 2 µm thickness were fabricated on a 250 nm thick non-stoichiometric amorphous silicon nitride (Si_xN_y) membrane (Edelman *et al.*, 1979; Löbl & Huppertz, 1998) of 2 mm x 2 mm in size, suspended on a silicon frame (6 mm x 6 mm). The 250 µm thick silicon wafer was processed as previously described (Opara *et al.*, 2017) by wet chemical etching using 20% KOH in water at 85°C to create freestanding Si_xN_y membranes. The pattern layout is divided into several areas, corresponding to marks of different shape and size (1 and 2 µm diameter disks and 3 µm wide crosses), and various spacing and arrangement (regular spacing of 20, 50 and 100 µm, and randomization of these positions). The main fabrication steps are pictured in Figure S1 and pattern layout details are given in Figure S2 in the Supporting Information.

The wafer with Si_xN_y membranes was initially cleaned with oxygen plasma (RIE, Oxford Instruments, 150 W forward power, 90 mTorr pressure, 30 sccm flow O_2, for 2 min). The chips with membranes were subsequently coated with Cr/Ge/Cr layers of respective thicknesses 5 nm/30 nm/5 nm in a Balzers BAK 600 evaporator (Evatec AG, Switzerland), to form a seed layer for electroplating. The growth rate of the layers was 0.1 nm/s for chromium and 0.23 nm/s for germanium, with the substrate placed 40 cm from the evaporation source. The upper Cr layer serves as an adhesion promoter for the PMMA

IMPORTANT: this document contains embedded data - to preserve data integrity, please ensure where possible that the IUCr Word tools (available from http://journals.iucr.org/services/docxtemplate/) are installed when editing this document.

structures. A PMMA (molecular mass: 950k, Microchem) layer was spincoated (Laurell spincoater WS-400-6NPP-LITE) from an anisole solution with spin speeds of 3500 rpm and 2000 rpm to reach thicknesses of at least 1 μm and 2 μm respectively. The samples were baked out for 15 min at 180°C on a hot plate and gradually cooled down to room temperature. The designed pattern was written in the PMMA layer by electron beam lithography with a Vistec EBPG 5000 PlusES machine using a dose of 3000 $\mu C/cm^2$, in so-called undersize-overdose conditions (Gorelick *et al.*, 2010). Subsequently, the PMMA layer was developed for about 20 s in a 3:7 mixture of H_2O of ultrapure quality (resistivity 18.2 MΩcm) and isopropanol (ultrapure, Technic, France), rinsed for 30 s in deionized H_2O, and dried using a flash of pressurized N_2. After development, the membranes were briefly cleaned with oxygen plasma (15 s with 150 W forward power, 30 sccm, 90 mTorr pressure). The upper chromium layer was removed with a 25 s chlorine plasma-etching step (CMA-4, BMP-Plasmatechnologie GmbH, Germany, mixure of 50 sccm O_2 and 20 sccm Cl_2). The gold structures were electroplated from a potassium gold cyanide ($K[Au(CN)_2]$) buffered electrolyte bath (Autronex™ GVC) of min. 68.1 wt. % of gold, and the nickel structures from a 185 g/l nickel sulphamate electrolyte solution (SEL-REX LECTRONIC 10-03 S, ERNE surface AG, Switzerland), as described by Hili *et al.* (Hili *et al.*, 2015). In the final step, the PMMA layer surrounding the electroplated pillars was removed by washing in acetone heated up to approx. 40°C.

The quality of the formed pillar structures was investigated by scanning electron microscopy at 5 keV accelerating voltage with a Zeiss Supra VP55 (Zeiss, Germany) equipped with both the in-lens and the Everhart-Thorneley secondary electron detectors.

S1.2. Preparation of fluorescent samples for the scanning maps

The model samples consisted of the gold or nickel structures described above. The frame surrounding the membrane carrying metal marks was inserted in the slit of a magnetic chip pin (Figure 3a), and glued with cyanoacrylate Cementit CA10 glue (merz+benteli AG, Niederwangen, Switzerland). Cementit glue can be dissolved in acetone for reusing the chip pins.

The representative samples, containing lipidic cubic phase (LCP) with embedded protein crystals, were prepared by sandwiching LCP containing crosslinked lysozyme microcrystals between Si_xN_y membranes. The LCP (about 20 μl total volume) was prepared by mixing monooleine (Nu-Check Prep, USA) and the aqueous phase in 60:40 ratio with coupled Hamilton syringes. The aqueous phase consisted of a suspension of lysozyme microcrystals, obtained by batch crystallisation by mixing 500 μl of 20 mg/ml lysozyme (Sigma-Aldrich) solution in 100 mM NaAc pH 3.0 and 500 μl of precipitant solution (68% dilution of the stock solution 28% NaCl, 8% PEG 6000, 100 mM NaAc pH 3.0), crosslinked by suspending the centrifuged crystals (2 min at 2600 g) overnight in a 2.5% solution glutaraldehyde. The size of the crystals ranged from 5 to 20 μm in size. For sandwiching, a mark-free 1000 nm-thick Si_xN_y membrane with a 5 mm x 5 mm frame and a 1.5 mm x 1.5 mm membrane (Silson,

Southam, United Kingdom) was fixed to a microscope slide. A double-sided sticky tape 140 µm spacer (MiTeGen, Ithaca, USA) cut to form a hole of the membrane size was stuck on top. A small LCP bolus was deposited on the flat membrane side, and the sandwich was closed by sticking a second Si_xN_y membrane on top, with the flat side facing the mesophase. The sandwich was separated from the glass slide and affixed to a standard protein crystallography sample pin with a double-sided sticky tape piece as described by Huang *et al.* (Huang *et al.*, 2016). The fluorescent marks were introduced in the representative protein-containing samples either by replacing the upper Si_xN_y membrane with a metal mark carrying membrane described above, or by replacing half of the aqueous phase with a 5% wt. suspension of steel microbeads (stainless steel metal microspheres 1-22 µm, SSMMS-7.8, Cospheric, Santa Barbara, USA) in milliQ water. The steel bead suspension was briefly sonicated in a bath to separate aggregates and agitated just before sampling to minimize bead decantation. Alternatively, instead of commercial Si_xN_y membranes, the steel bead samples were prepared between two 25-µm thick cyclic olefin copolymer (COC) films, following the IMISX setup reported by Huang *et al.* (Huang *et al.*, 2016). The samples were snap-cooled in liquid nitrogen. Micrographs of the samples prior to snap-cooling are presented in Figure S3.

S1.3. Data processing

The raw data from the JUNGFRAU detector was processed with the standard correction algorithm presented by Redford *et al.* (Redford *et al.*, 2018). Due to the low incoming flux, the automatic gain switching feature was never engaged and all pixels remained in the highest gain. The pedestal raw analog-to-digital converter (ADC) output from the pixel matrix was determined in a dedicated dark measurement, and subtracted pixel by pixel from the raw ADC pixel matrix output. Then the result was corrected with a pixel-wise gain correction factor determined independently, to obtain the measured energy per pixel (Redford *et al.*, 2018).

Due to the critical dependence of this measurement to the stability of the recorded energy, at the SLS a tracking procedure to follow slow drifts in the pedestal values was applied to the data (Redford *et al.*, 2018): a subsample of empty pixels was used in each frame to track the pedestal. The peak around zero of this distribution, corresponding to pixels without photon hits, was fitted with a Gaussian curve, and the mean value of said curve was used to obtain a global value to correct for pedestal shifts. This is a simple and effective way to correct for common mode fluctuations (which were generally small) and also (stronger) global temperature/sensor effects. At SwissFEL this tracking procedure is not necessary thanks to the short integration time.

Further processing was performed using *ROOT* (Brun & Rademakers, 1997) and customized python scripts. In selected cases, a clustering procedure was carried out to correct for charge-sharing effects between neighboring pixels, when a photon hits the corner or edge of a pixel. On the pedestal subtracted and gain corrected 2D-array, square 4-pixel clusters were selected around local maxima, over which the

energies were summed. After photon energy extraction, the cluster was set to zero to avoid a new cluster being found on the same pixels while analyzing the remaining of the 2D-array.

The measured energy values of all pixels (or pixel clusters) within a defined spatial region of interest on the detector were used to compute a histogram showing the number of pixels having received a given energy within the exposure, cumulative on all images corresponding to a grid cell. Examples of spectra obtained are shown in Figure 3b. For each metal element, counts were integrated within a region of interest in energy (ROI) corresponding to the fluorescence energy of the metal: 8.5-10.3 keV for Au, 6.2-8.2 keV for Ni and 5.3-7.2 keV for Fe. The ROI counts for each grid cell are displayed as fluorescence maps. In the case of the XFEL measurements, the Ni ROI counts were normalized by the counts in a direct beam ROI (8.3-9.3 keV), to eliminate pulse-to-pulse variations of the intensity in SASE mode. Signal-over-background ratios (SBR) were calculated by dividing average counts in the few cells of the map with high counts (where metal is present) by average counts from a large area of the map with low counts (where metal is absent). Standard deviations on the SBR were calculated using SBRs either from several different metal marks in the same map at the SLS, or from several independent measurements of the same mark at SwissFEL. Briefly, for EIGER 16M scanning maps, pixel counts were summed over a defined area of the detector (a square area centered around the beam center, covering the same solid angle as the JUNGFRAU 1M geometry, see Supporting Information Figure S10).

Dose calculations were performed using RADDOSE-3D v1.2.467 (Zeldin *et al.*, 2013), not using any particular heavy atom content, but a safety factor was included by considering the maximum beamline flux of $1 \cdot 10^{12}$ photons$\cdot$s^{-1}.

The distance measurements between metal marks were carried out either by taking horizontal and vertical slices in the map, or by projecting the signal from each mark in both scanning directions by summing all rows or columns in the mark area. A calculated overlap model was then fitted to the experimental signal by scale optimization and translation (0.05 µm steps). The deviation from the theoretical distance was calculated as the absolute value of the difference between the measured distance and the distance on the photolithography mask pattern design. In the oversampled maps, a small offset was introduced every other line to account for a small triggering delay in the rastering setup (see Figure S7 in the Supporting Information).

S2. Absorption edge scan experiments

S2.1. Material and methods

S2.1.1. Selenium-containing crystal

Crystals were kindly provided by Jérôme Basquin (Max Planck Institute of Biochemistry, Martinsried). The selenomethionine substituted CWC22-CWC27-EIF4A3 complex was set up for crystallization at 20 mg/ml in SEC buffer (20 mm Tris pH 7.4,250 mM NaCl, 2mM DTT) by sitting-drop vapor diffusion

in 0.2 uL drops obtained by mixture of equal volumes of protein and crystallization solution. Crystals appeared after 2 days at 4°C as monoclinic prism after mixing with 20% (w/v) PEG20000, 50 mM MES pH 6.5 and were cryoprotected in reservoir solution containing 33% (v/v) ethylene glycol prior to flash freezing in liquid nitrogen. The protein crystallized in the $P2_12_12_1$ space group with cell parameters of a=152 Å, b=165 Å, c=181 Å, α=β=γ=90°. The asymmetric unit contained 4 molecules of the complex, with 12 Se atoms per molecule (for 514 amino-acids in the sequence).

S2.1.2. Mercury-soaked lysozyme

Chicken egg white Lysozyme from Sigma Aldrich (10837059001) was dissolved at a concentration of 50mg/mL in 50mM Na Acetate pH 4.5. The crystallisation experiment was carried out in a CrysChem sitting drop vapor diffusion plate with protein drops of 2uL and 2uL of precipitant against 500uL of precipitant consisting of 5% PEG MME 5000, 2M NaCl, 50mM Na Acetate pH 4.5, 25% ethylene glycol. The plate was stored at 20°C, crystals appeared overnight and kept growing for 3 days. A 1uL drop of Mercury(II) acetate dissolved at 10mM in water was added to the crystallisation drops 10 minutes before cryo-cooling crystals in liquid nitrogen.

S2.1.3. Data collection and analysis

For the absorption edge scanning experiments, the beam transmission was set to 5% for Se and 0.5% for Hg. The Si(111) monochromator energy was stepped by 5 eV or 2 eV increments depending on the distance to the theoretical edge. At each energy reached by the monochromator, a series of 5000 images for Se, or of 9000 images for Hg was recorded at 2.2 kHz repetition rate with a 50 µs integration time, but reliable results were obtained already using the first 1000, respectively 3000 images, corresponding respectively to 0.45 s and 1.4 s total shutter opening time at each energy. The sample-to-detector distance was of 55 mm and the sample-to-beamstop distance was of 15 mm. The beam was defocused to a size of 50 µm x 50 µm.

For the absorption edge scanning experiment, energy histograms were calculated over the 2 furthest corners of the detector from the beamstop, between -2 and 30 keV with 500 bins. The spectra obtained are shown in Figure S8 in the Supporting Information. The counts in the metal ROI (9 to 10 keV for Hg and 10-11 keV for Se) were normalized by the maximum of counts at the direct beam energy to account for the beam intensity decrease caused by the absence of beam optimization during energy scanning. The program *chooch* (Evans & Pettifer, 2001) was used for obtaining anomalous scattering coefficients f' and f'' from the relative fluorescence signal. For comparison, standard edge scan data was recorded on the exact same selenomethionine crystal using a typical beamline configuration with the beamline-integrated SDD (Ketek AXIS) placed in backscattering geometry with an angle of 45° (Fuchs *et al.*, 2014), at beamlines X06SA and X06DA, using a transmission of 1% and 1s exposure time at each energy.

S2.2. Results

Representative data for the absorption edge scans is given in Figure S8, using 1000 images for Se and 3000 images for Hg. Adding more of the recorded images resulted in smoother curves, but no major change in edge profile and *chooch* results. The absence of a peak around 25 keV in Figure S8A shows that each pixel received at most one photon during the integration time. JF spectra display wider peaks than SDD spectra due to electronic noise, and a continuous background from charge sharing is observed.

Table S1 shows the results from *chooch* for the Se-containing sample. Peak and inflection energies are within 1 eV from the determinations using an SDD at 2 different beamlines, which is lower than the typical energy resolution of the beamlines. The values of the scattering coefficients f' and f'' are within 2 electrons, which is comparable to the difference observed between SDD measurements at 2 different beamlines. We conclude that the edges scan measurement is of similar quality compared to the SDD measurements, in spite of the reduced sharpness of the obtained spectra compared to SDD spectra.

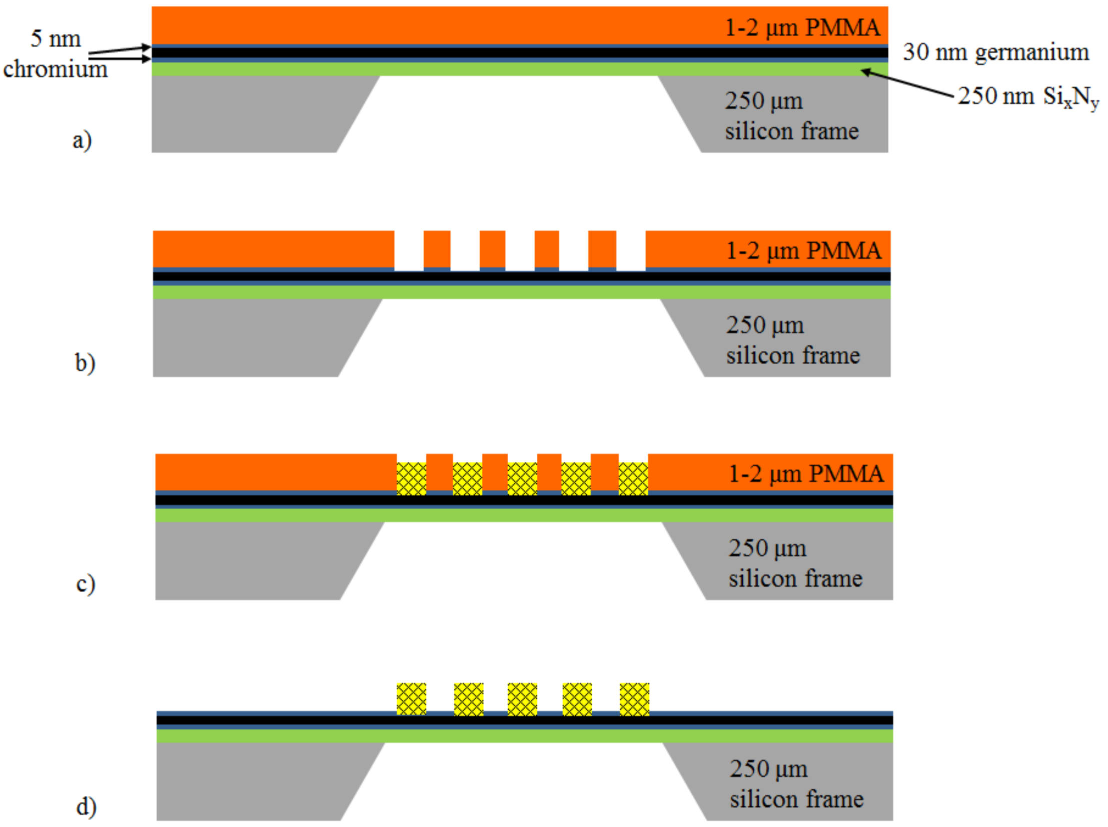

Figure S1 Scheme of metal mark fabrication on Si_xN_y membranes by electroplating: a) Si_xN_y membrane over a silicon frame covered with a stack of 5 nm Cr, 30 nm Ge, 5 nm Cr and 1-2 µm PMMA, *cf.* Materials and Methods section, b) e-beam exposure, development and opening of the chromium layer c) electroplating the structures in the PMMA mold, filling of the formed cavities with

metal, d) PMMA removal in the acetone bath, leaving the formed marks on the freestanding Si_xN_y membrane.

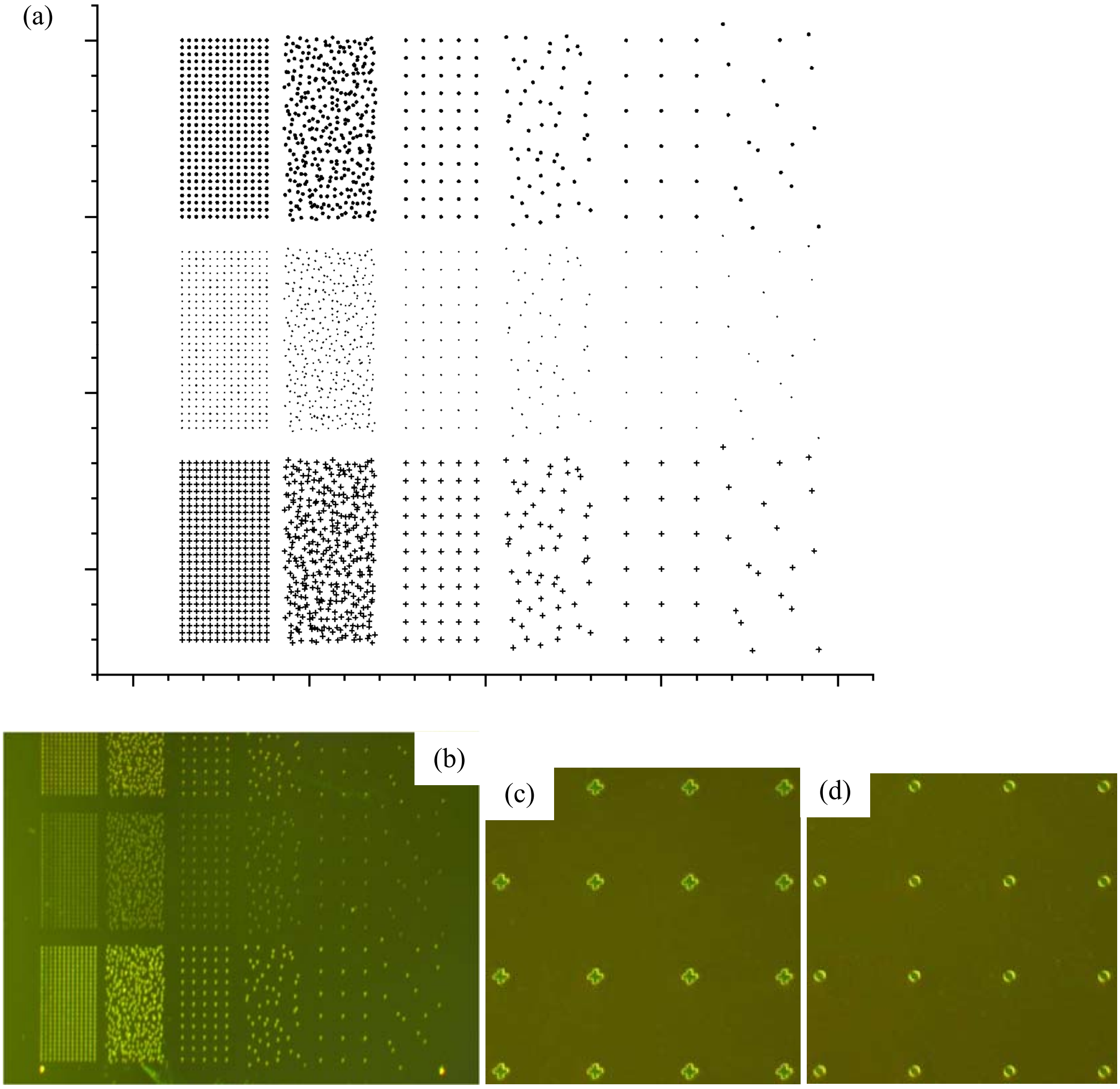

Figure S2 (a) Layout of the electroplated model metal samples. For visibility, the marks are drawn larger than their actual size. The pattern is divided in 9 zones in a 3 by 3 layout. Each zone contains a set of regular marks on the left and randomly distributed marks on the right. The randomly distributed marks were obtained by randomizing the positions of the regular arrays within the same surface area. In the upper 3 zones, marks were 2 µm wide disks. In the 3 zones in the middle (horizontally), marks were 1 µm disks. In the bottom 3 zones, marks were 3 µm wide crosses. The spacing between marks in the regular arrays was of 20 µm on the left, 50 µm in the middle (vertically), and 100 µm on the right. In the figure, the major ticks are spaced out by 500 µm, and the minor ticks by 100 µm. (b) Optical micrographs of a metal sample, with close-ups on the regular 20 µm spaced crosses area (c) and 2 µm circles area (d).

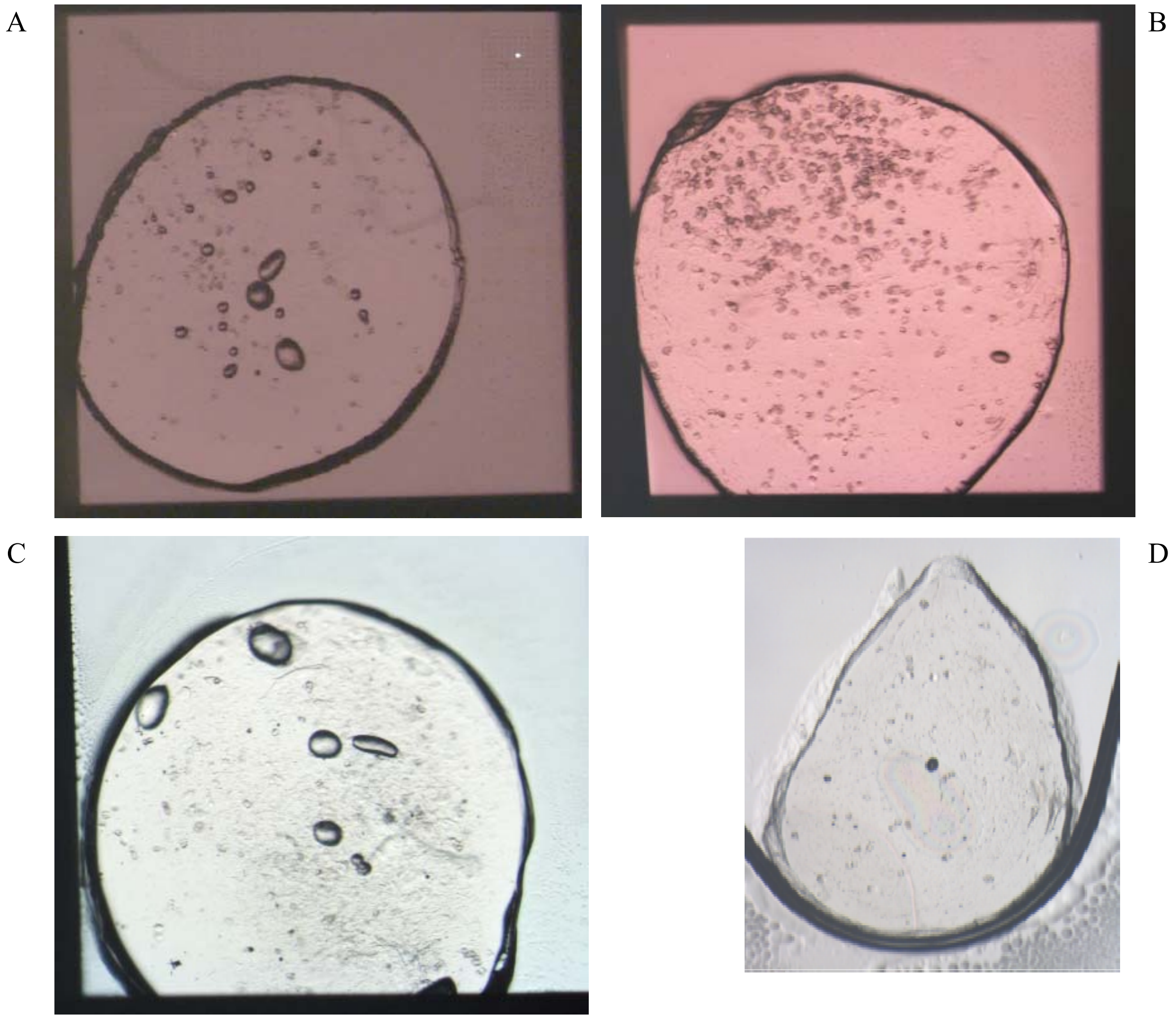

Figure S3 Optical microscopy pictures of the samples with the protein crystal containing LCP media prior to snap-cooling: (A) Si_xN_y sandwich with gold marks, (B) Si_xN_y sandwich with nickel marks, (C) Si_xN_y sandwich with steel beads, (D) COC sandwich with steel beads. For scale, the size of the Si_xN_y window, visible as a black frame in A-C, is 2 mm x 2 mm, and picture D is taken at the same magnification.

A

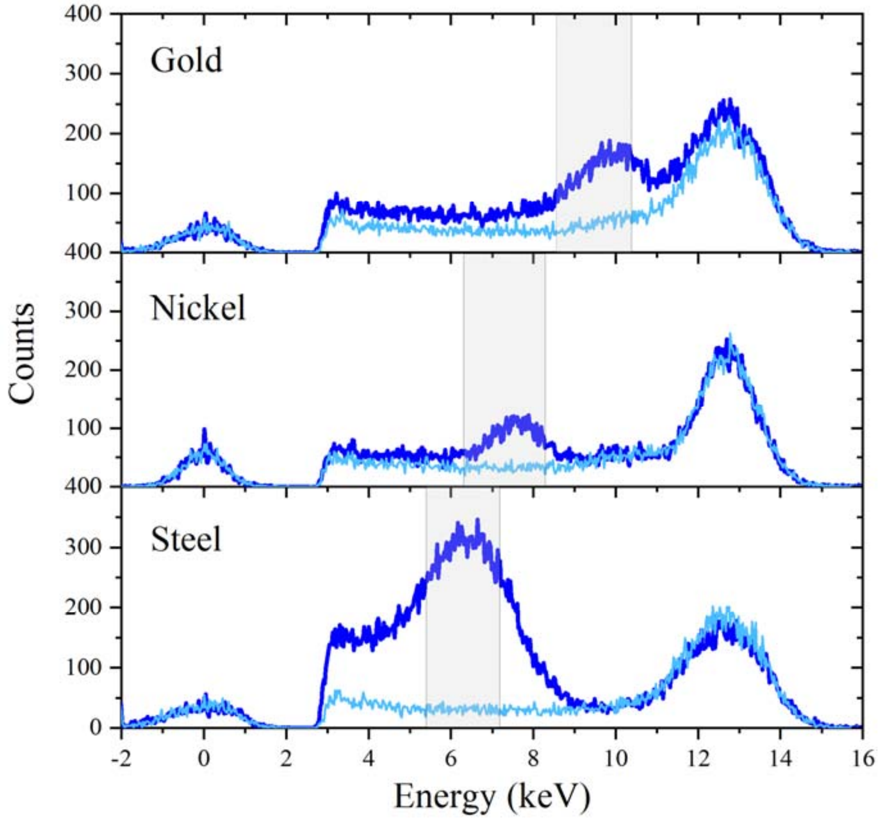

B

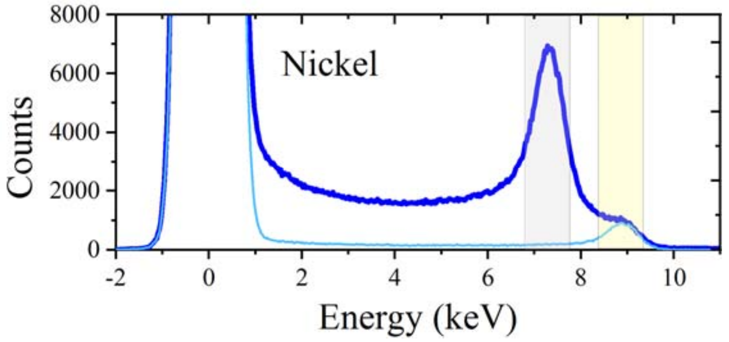

Figure S4 Full range represnetative spectra from the fluorescence maps obtained at SLS (A) and SwissFEL (B), corresponding to the curves shown respectively in Figure 3b and d of the main manuscript. The additional peak centered around 0 keV is the noise peak from the detector, and contains no information from the sample. In the case of the SLS data, the signal below 3 keV has been filtered out to 1% for better visibility and use in pedestal tracking.

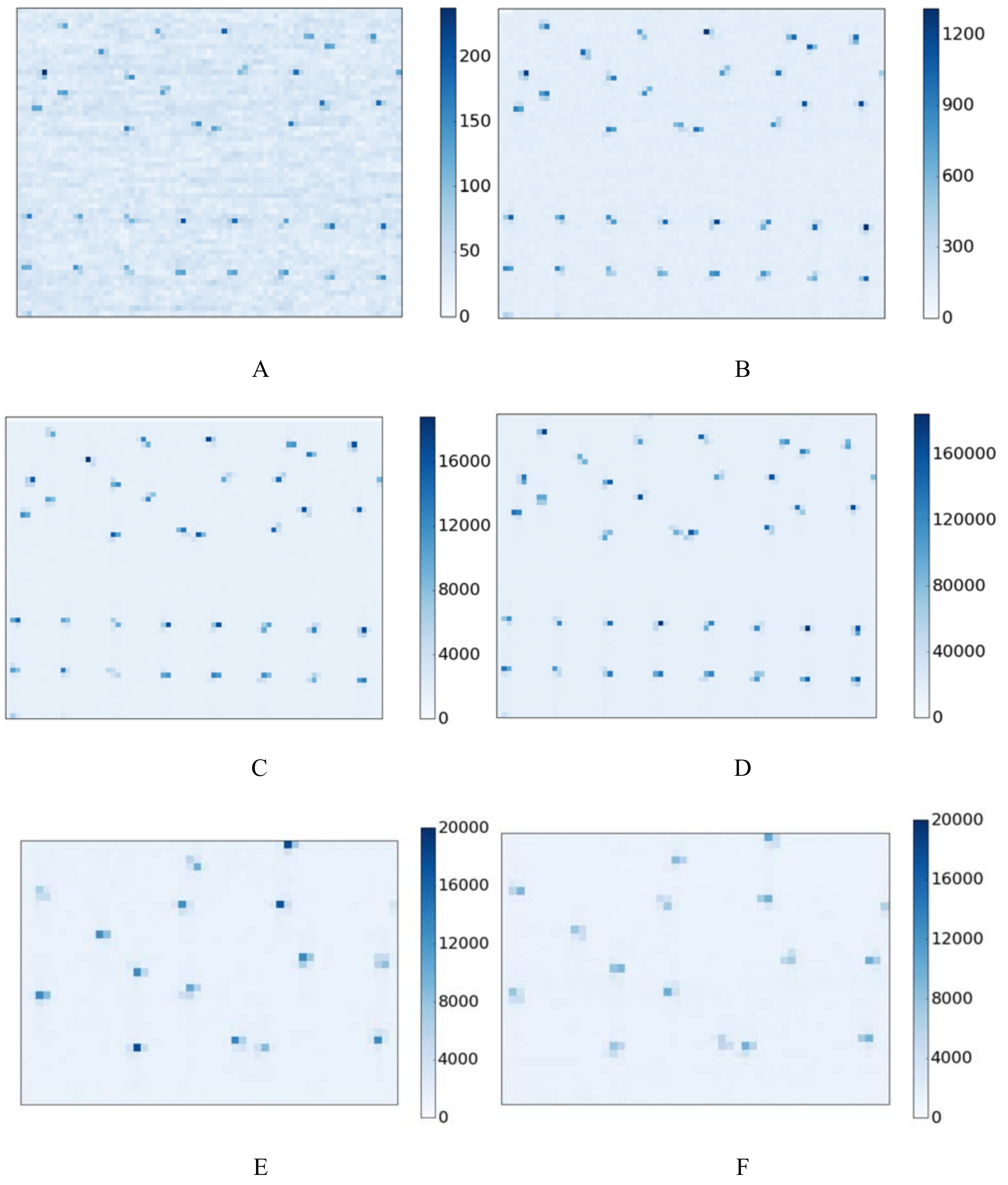

Figure S5 Fluorescence maps obtained from gold model samples while varying the transmission: 0.04% (A), 0.4% (B), 4% (C) and 40% (D), and while varying the rastering speed: 100 Hz-10 images/cell (E) and 10 Hz-100 images/cell (F). E and F are plotted on the same scale.

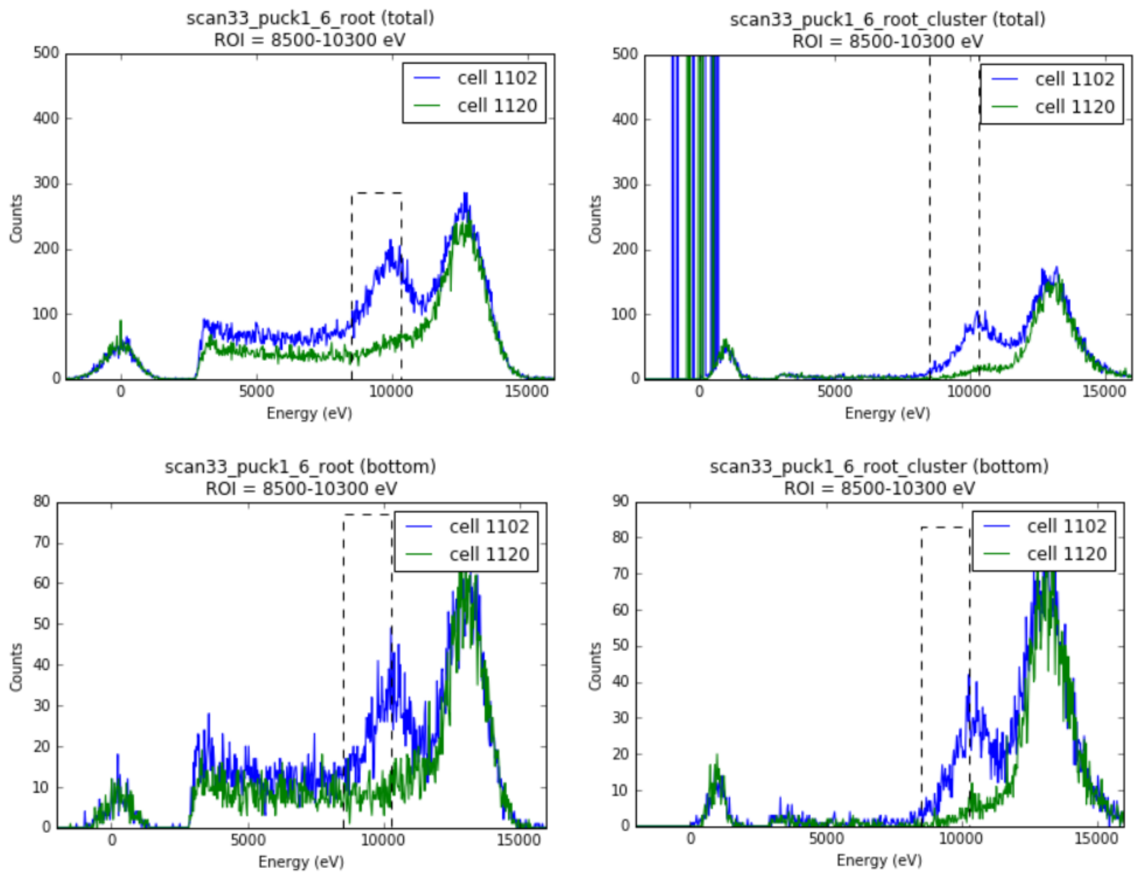

Figure S6 Representative spectra from a gold real sample (Si_xN_y sandwich), obtained by using the full detector area (top) versus only the lower half of the lower module (bottom), i.e. only a quarter of the detector area. The left column shows spectra obtained using individual pixels information, while the right column shows spectra obtained by clustering. The spectra from 2 different cells in a fluorescence map grid are shown. Cell 1102 is a cell where the fluorescence intensity was maximum, and cell 1120 had no metal present.

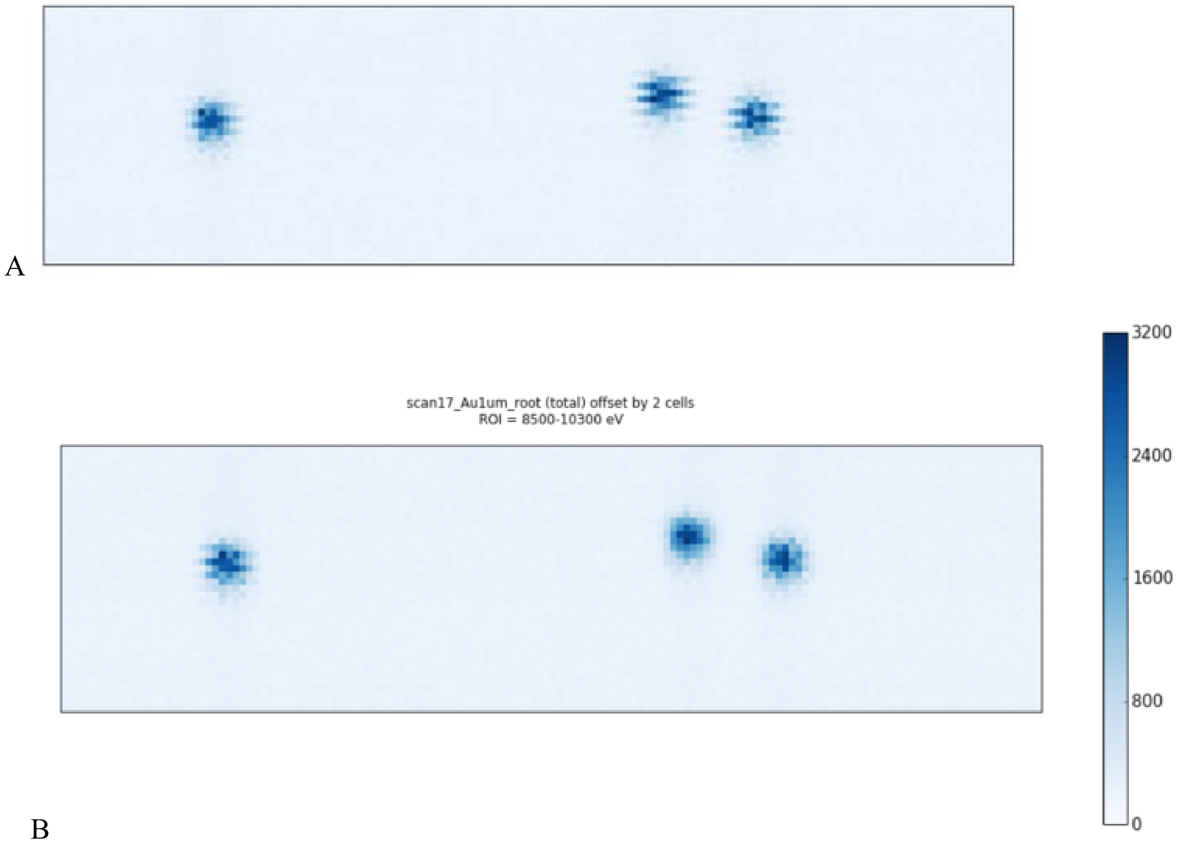

Figure S7 Oversampled maps with 1 μm cell size, before (A) and after (B) the offset correction. This offset correction consisted in offsetting every other line by 2 cells, in order to compensate for a systematic triggering error.

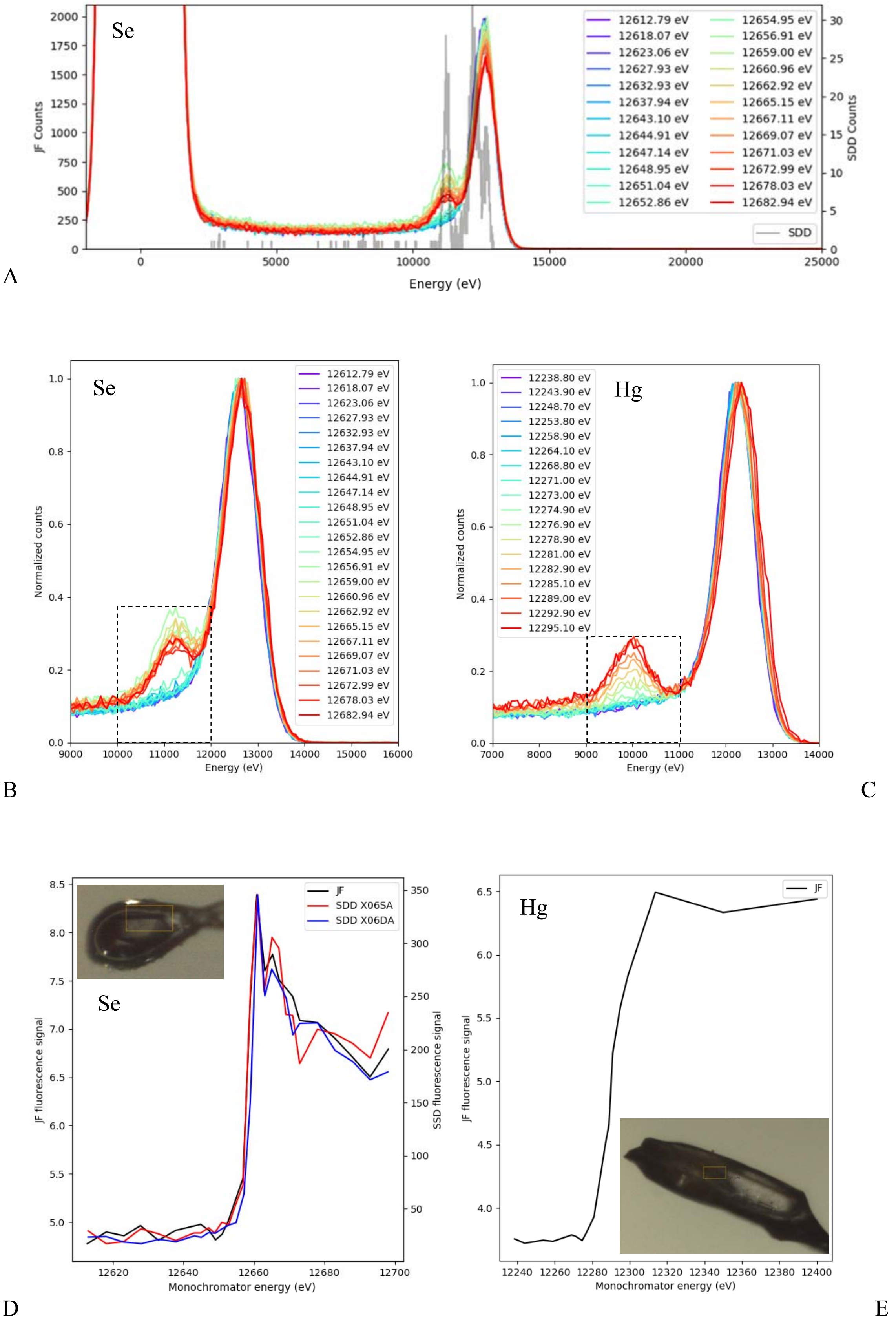

Figure S8 Absorption edge scans: (A) Raw histograms from the Se edge scan for the different monochromator energies, in comparison to an SDD spectrum taken above the edge. (B, C) Portions of the histograms normalized to the high of the direct beam energy peak, Se in B, Hg in C. The integration

range is shown as a dashed line. (D) JUNGFRAU edge profile for the Se-containing sample, compared to edges measured on the same sample with SDDs at the X06SA and X06DA beamlines of the SLS. (E) JUNGFRAU edge profile for the Hg-containing sample. Insets in (D, E) show the crystals measured. The yellow box is 90 µm x 50 µm. 1000 images were used for Se, 3000 images for Hg.

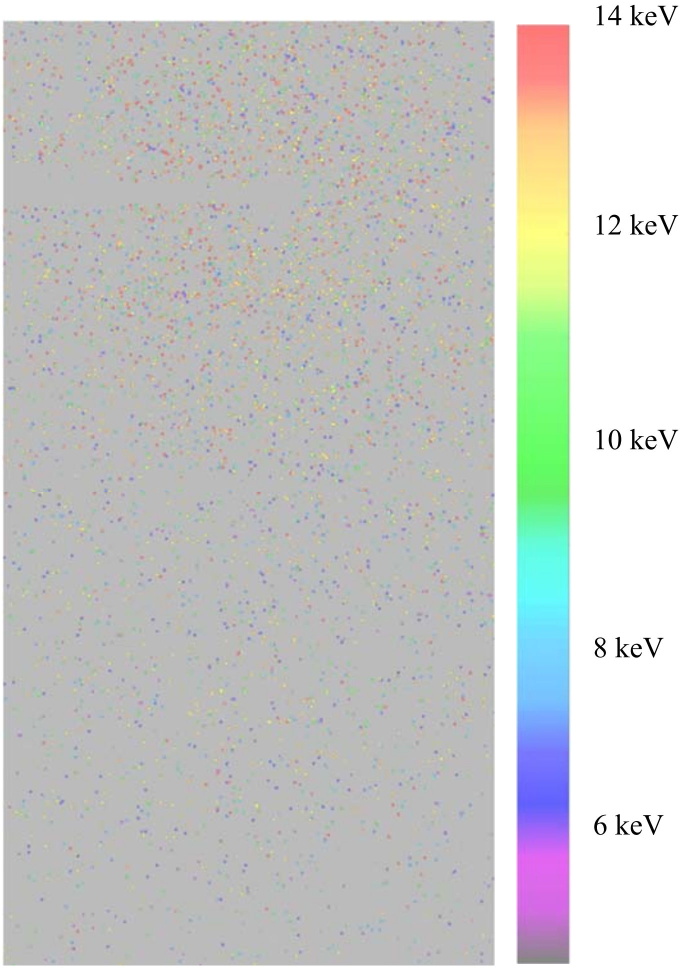

Figure S9 Example of readout image from the JUNGRAU detector, recorded on a fluorescent nickel mark with 2 % transmission: 20 images were summed (to cover the complete cell), with 2.5 keV threshold. Only a about 1/8 of the detector is shown, from beam center to detector edge, for visibility of the small pixels. The beamstop shadow is visible in the upper part.

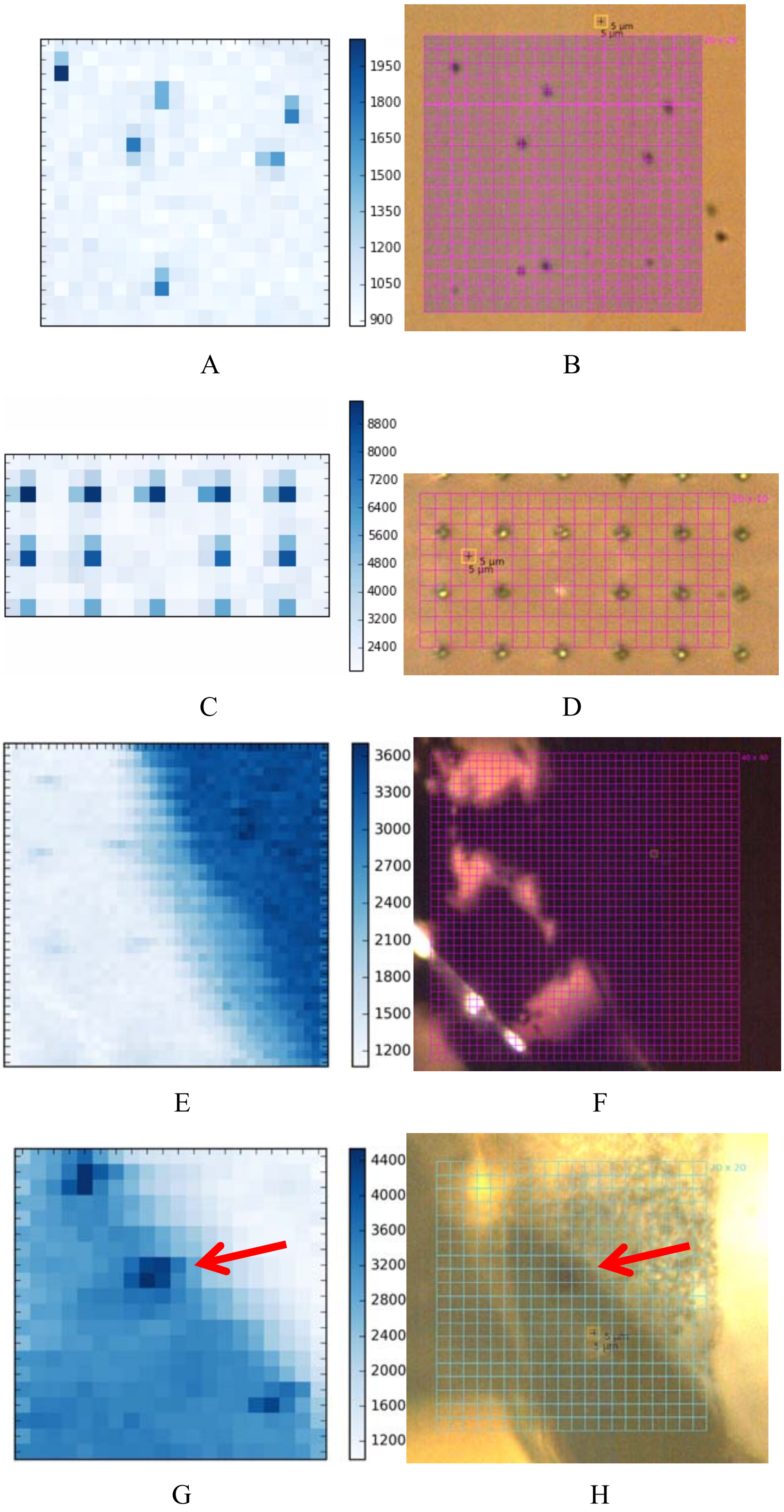

Figure S10 Examples of EIGER 16M maps on model samples (A, C) and real samples (E, G) based on total counts summed over a large detector area, a square of 3376 pixels size centered on the beam center. The exposure was 0.1 s (10 Hz rastering), and the beam transmission was 0.01% for the model samples, and 0.01 s (100 Hz rastering) and 0.1% transmission for the real-life samples, i.e. the number of photons per cell is identical to the JUNGFRAU reference maps, and between scans. For the nickel

model sample (A), the SBR obtained was 1.7 ± 0.2. For the gold sample (C), the SBR was 4.2 ± 0.1. (B, D, F, H) Corresponding areas on the model and real samples. For the gold real sample (E), the scanning area overlaps the LCP bolus on the right side. The marks are detectable only in the left area, where no LCP is present. For the real sample with steel beads mixed in LCP and enclosed in a Si_xN_y sandwich, the red arrow shows the only effectively present steel bead in the scanning area. Other high-counts features are artifacts illustrating the unreliability of the detection with EIGER in presence of LCP.

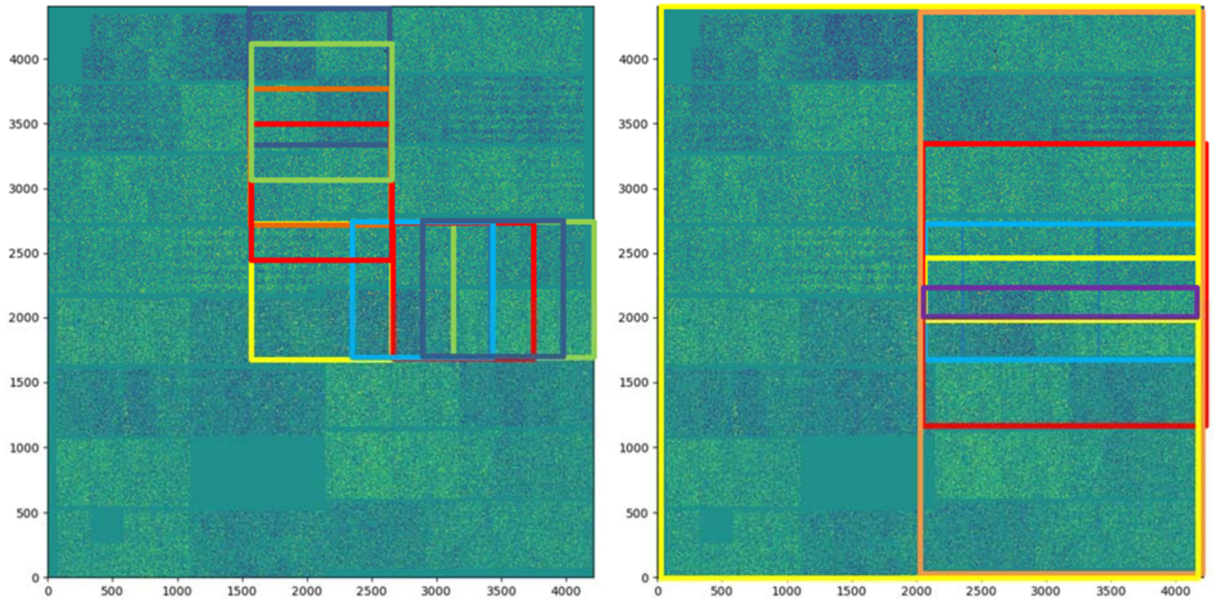

(a) (b)

Figure S11 Spatial ROIs used on the JUNGFRAU 16M for the SwissFEL data analysis, viewed in the direction opposite to the beam propagation. The right and top areas were predominantly used because of the absence of any bad module or shadow from devices such as the cryojet and post-sample tube. (a) 1M ROIs with the vertical and horizontal offset used for Figure 4b in the main manuscript. (b) ROIs of variable size used for Figure 4c. For clarity, ROIs smaller than 0.5M are not shown, and the rectangles are very slightly offset for visibility of their overlapping edges.

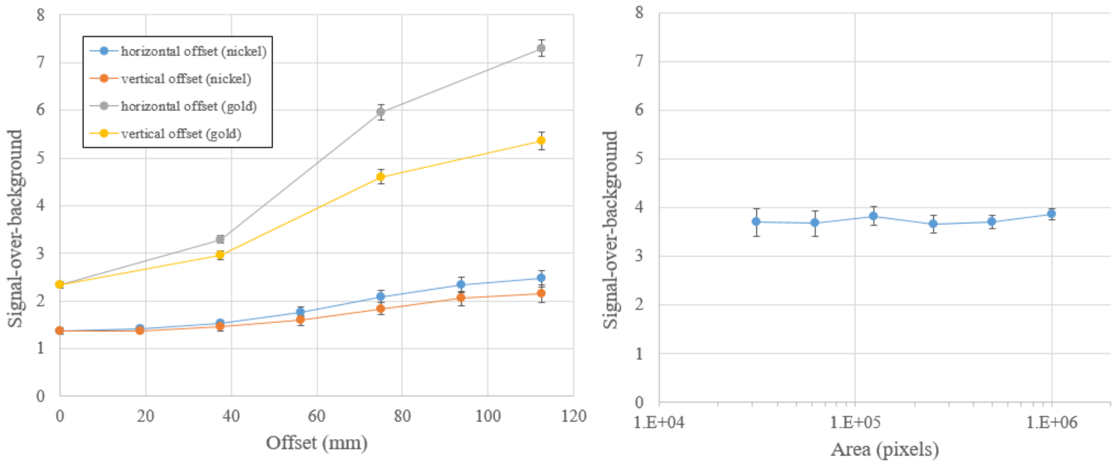

(a) (b)

Figure S12 Results from measurements with the EIGER 16M detector, using total counts on regions of interest defined similarly as in Figure S11. (a) SBR as a function of the offset horizontally and vertically to the beam center for gold and nickel model samples, using a square 1 Mpix ROI. The SBR depends highly on the position of the ROI. At larger offsets, the SNB becomes higher thanks to the isotropy of fluorescence. At low offsets, the SBR decreases and makes the detection difficult. The separation between vertical and horizontal offsets arises from the beam polarization. (b) SBR as a function of the area of the ROI, for the nickel model sample. The SBR is maintained even for small ROIs, but the standard error increases.

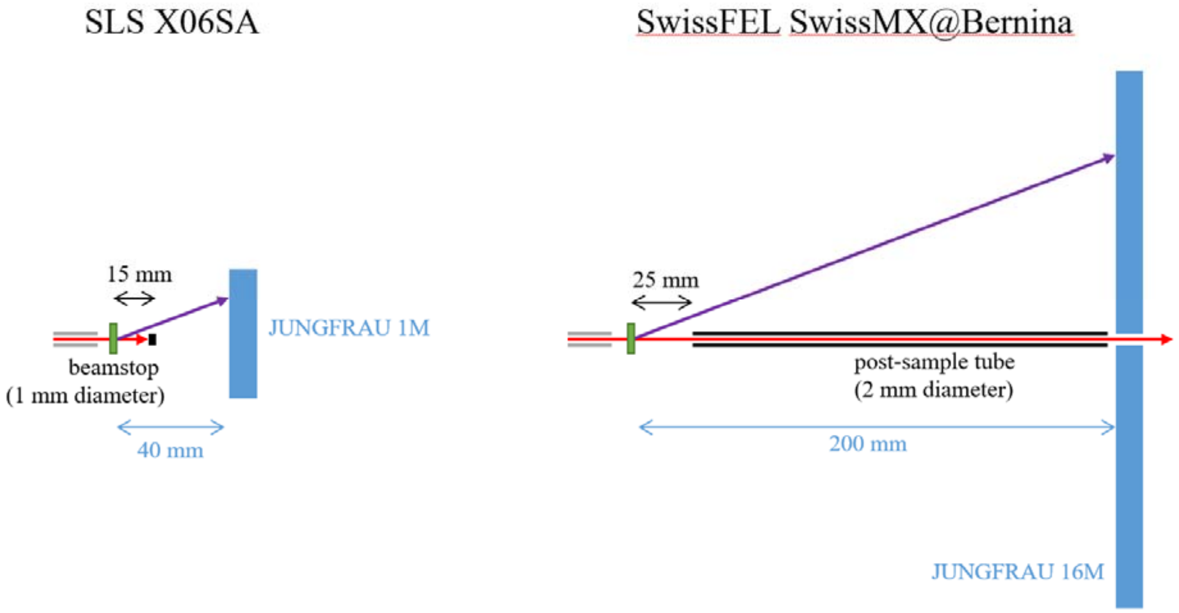

Figure S13 Schematics of the experimental setups with relevant distances noted. The direct X-ray beam is shown in red, a diffracted ray in purple. Not indicated for clarity is the collimator (grey) to sample (green) distance, of about 4 mm in both cases. Schematics are approximately to scale.

Table S1 Edge scan results for Se, obtained using 1000 images

		Energy (eV)	f'' (e)	f' (e)
**JUNGFRAU**	**Peak**	12660.80	5.98	-7.88
	**Inflection**	12659.00	4.38	-9.66
**SDD X06SA**	**Peak**	12661.18	5.25	-7.48
	**Inflection**	12658.35	2.88	-9.34
**SDD X06DA**	**Peak**	12661.34	4.65	-7.49
	**Inflection**	12659.58	2.80	-8.85

Table S2 Edge scan results for Hg, obtained using 3000 images

		Energy (eV)	f'' (e)	f' (e)
**JUNGFRAU**	**Peak**	12313.80	9.87	-13.44
	**Inflection**	12291.00	7.19	-16.17

References

Brun, R. & Rademakers, F. (1997). *Nucl. Instruments Methods Phys. Res. Sect. A Accel. Spectrometers, Detect. Assoc. Equip.* **389**, 81–86.

Edelman, F. L., Zaitsev, B. N., Latuta, V. Z. & Khoromenko, A. A. (1979). *Phys. Status Solidi*. **51**, 49–56.

Evans, G. & Pettifer, R. F. (2001). *J. Appl. Crystallogr.* **34**, 82–86.

Fuchs, M. R., Pradervand, C., Thominet, V., Schneider, R., Panepucci, E., Grunder, M., Gabadinho, J., Dworkowski, F. S. N., Tomizaki, T., Schneider, J., Mayer, A., Curtin, A., Olieric, V., Frommherz, U., Kotrle, G., Welte, J., Wang, X., Maag, S., Schulze-Briese, C. & Wang, M. (2014). *J. Synchrotron Radiat.* **21**, 340–351.

Gorelick, S., Guzenko, V. A., Vila-Comamala, J. & David, C. (2010). *Nanotechnology*. **21**, 295303.

Hili, K., Fan, D., Guzenko, V. A. & Ekinci, Y. (2015). *Microelectron. Eng.* **141**, 122–128.

Huang, C.-Y., Olieric, V., Ma, P., Howe, N., Vogeley, L., Liu, X., Warshamanage, R., Weinert, T., Panepucci, E., Kobilka, B., Diederichs, K., Wang, M. & Caffrey, M. (2016). *Acta Crystallogr. Sect. D Struct. Biol.* **72**, 93–112.

Löbl, H. . & Huppertz, M. (1998). *Thin Solid Films*. **317**, 153–156.

Opara, N., Martiel, I., Arnold, S. A., Braun, T., Stahlberg, H., Makita, M., David, C. & Padeste, C. (2017). *J. Appl. Crystallogr.* **50**, 909–918.

Redford, S., Andrä, M., Barten, R., Bergamaschi, A., Brückner, M., Chiriotti, S., Dinapoli, R., Fröjdh, E., Greiffenberg, D., Leonarski, F., Lopez-Cuenca, C., Mezza, D., Mozzanica, A., Ruder, C., Schmitt, B., Shi, X., Thattil, D., Tinti, G., Vetter, S. & Zhang, J. (2018). *J. Instrum.* **13**, C11006–C11006.

Zeldin, O. B., Gerstel, M. & Garman, E. F. (2013). *J. Appl. Crystallogr.* **46**, 1225–1230.
